# KRT14 Drives Basal Muscle‐Invasive Bladder Cancer Progression and Lung Metastasis by Directly Binding to and Stabilizing IGF2BP1

**DOI:** 10.1002/advs.75900

**Published:** 2026-06-09

**Authors:** Shirui Huang, Zhihan Zhou, Qipeng Xie, Zheng Wang, Lijiong He, Yutong Liu, Lijuan Huang, Liuxian Ye, Limeng Hu, Baokun Li, Yang Wang, Xiaohui Hua, Xuelei Liu, Yunping Zhao, Jingxia Li, Jinfei Chen, Guiying Wang, Xian Shen, Wei Chen, Chuanshu Huang

**Affiliations:** ^1^ Key Laboratory of Medicine Ministry of Education School of Laboratory Medicine and Life Sciences Wenzhou Medical University Wenzhou Zhejiang China; ^2^ Oujiang Laboratory Zhejiang Lab for Regenerative Medicine, Vision and Brain Health Wenzhou Zhejiang China; ^3^ The Department of General Surgery the Second Hospital of Hebei Medical University Shijiazhuang Hebei China; ^4^ The Second Department of Surgery the Fourth Hospital of Hebei Medical University Shijiazhuang Hebei China; ^5^ Center of Biomedical Physics Wenzhou Institute University of Chinese Academy of Sciences Wenzhou Zhejiang China; ^6^ Department of Occupational Health and Environmental Health School of Public Health Anhui Medical University Hefei Anhui China; ^7^ Department of Oncology The First Affiliated Hospital of Wenzhou Medical University Wenzhou Zhejiang China; ^8^ Department of General Surgery The First Affiliated Hospital of Wenzhou Medical University Wenzhou Zhejiang China; ^9^ Department of Urology The First Affiliated Hospital of Wenzhou Medical University Wenzhou Zhejiang China

**Keywords:** Basal‐type muscle invasive bladder cancer, IGF2BP1, KRT14, metastasis, RNA localization

## Abstract

Basal‐type muscle‐invasive bladder cancer (BMIBC) is characterized by aggressive metastasis and poor prognosis but lacks molecularly defined therapeutic targets. Through integrative analyses of clinical cohorts and BBN‐induced mouse models, we identified KRT14 as a core oncogenic driver that orchestrates the KRT14‐IGF2BP1 signaling axis to promote BMIBC progression and lung metastasis. Mechanistically, residues K294 and E295 within the KH2 domain of IGF2BP1 specifically recognize conserved residues D226 and E227 within the nuclear export signal of KRT14. This direct interaction facilitates IGF2BP1‐mediated cytoplasmic trafficking and auto‐stabilization of its own mRNA, establishing a positive feedback loop that amplifies IGF2BP1‐targeted pro‐invasive gene expression. Functional disruption of this axis suppressed primary tumor progression and lung metastasis in BMIBC models. Collectively, our findings define the KRT14‐IGF2BP1 axis as a previously unrecognized, potentially targetable vulnerability in BMIBC, whose inhibition may limit aggressive disease progression and inform future therapeutic strategies.

## Graphical abstract

We identified a *Krt14^+^ Igf2bp1^+^* bladder cancer population linked to EMT. Mechanistically, KRT14 stabilizes and transports IGF2BP1 to invasive protrusions, initiating a positive feedback loop that drives metastasis and poor outcomes.

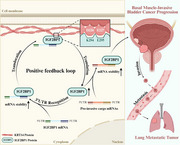

## 1 Introduction

Muscle‐invasive bladder cancer (MIBC) represents a highly aggressive and lethal subtype of bladder cancer (BCa), accounting for approximately 25–30% of all cases. It is associated with a high risk of progression, distant metastasis, and cancer‐related mortality [1, 2]. Without treatment, the 5‐year survival rate can be as low as 5% [3]. Molecular classification divides MIBC into luminal and basal subtypes, among which BMIBC is characterized by the most aggressive biological behavior and the poorest prognosis [4, 5]. The current standard treatment, radical cystectomy combined with cisplatin‐based neoadjuvant chemotherapy [6], results in the permanent loss of bladder function and is associated with substantial postoperative complications, leading to significantly reduced quality of life [7]. Therefore, identifying precise molecular targets and developing effective therapeutic strategies for BMIBC are critical to improving clinical outcomes.

In BCa, tumors characterized by KRT5/6^+^, KRT14^+^, FOXA1^−^, and GATA3^−^ expression (where ^+^ and ^–^ denote positive and negative marker expression, respectively) are classified as BMIBC [8]. Among these markers, KRT14, a type I acidic keratin, serves as a canonical hallmark of basal‐like subtypes across multiple malignancies, including bladder [4], breast [9, 10], and pancreatic cancer [11]. KRT14‐high tumors in bladder and breast cancer show significantly worse patient survival [12, 13], whereas in pancreatic cancer, KRT14 marks the basal‐like subtype that is associated with poor prognosis [14]. Moreover, *Krt14* ^+^ cells have been implicated in bladder tissue repair and tumorigenesis [15]. Despite its utility as a biomarker, whether KRT14 functionally drives BMIBC progression and metastasis, and the molecular mechanisms underlying this process, remain poorly understood.

Here, we identify KRT14 as a key driver of BMIBC malignancy and a potential therapeutic target. To resolve intratumoral heterogeneity and delineate cellular programs underlying tumor aggressiveness, we performed single‐cell RNA sequencing (scRNA‐seq) on patient‐derived tumors. This analysis revealed that KRT14 is highly expressed in epithelial subpopulations exhibiting pronounced epithelial‐mesenchymal transition (EMT) and cancer stem cell (CSC) signatures, suggesting its enrichment within highly aggressive tumor populations. To further substantiate this finding, cross‐species scRNA‐seq analyses of human BCa and BBN‐induced mouse tumors demonstrated that KRT14 expression was markedly elevated in tumor epithelium compared with normal urothelium. Moreover, in the BBN‐induced BMIBC model, both the proportion and expression level of *Krt14^+^* epithelial cells progressively increased during tumor development, consistent with a role for KRT14 in promoting a hybrid epithelial/mesenchymal (E/M) phenotype while preserving epithelial identity. Collectively, these findings suggest that KRT14 is a conserved epithelial factor closely associated with the aggressive progression of BMIBC.

Building on these findings, we next investigated the clinical significance and functional impact of KRT14 upregulation in BMIBC. Integrative analyses of independent datasets and clinical specimens consistently demonstrated that elevated KRT14 expression correlated with poor prognosis. Using a BBN‐induced Krt14 conditional knockout (CKO) mouse model, we found that loss of Krt14 markedly attenuated BMIBC progression. ScRNA‐seq of bladder tissues from BBN‐induced Krt14‐CKO mice revealed an almost complete depletion of *Krt14* ^+^ *Igf2bp1* ^+^ basal and EMT‐like cells. Pseudotime trajectory analysis further delineated a developmental trajectory from *Krt14* ^+^ *Igf2bp1* ^+^ basal cells toward a stable hybrid E/M state, providing in vivo evidence that the KRT14‐IGF2BP1 axis underlies BMIBC dissemination without a complete loss of epithelial identity. Mechanistically, KRT14 facilitates BMIBC progression through two coordinated processes: (1) enhancing IGF2BP1 binding to and stabilization of its own mRNA, thereby establishing a post‐transcriptional positive feedback loop that amplifies IGF2BP1 protein expression, and (2) promoting the peripheral localization of IGF2BP1 mRNA and IGF2BP1‐associated pro‐invasive cargo transcripts, thereby sustaining hybrid E/M gene networks associated with epithelial plasticity and enhancing the invasion capacity of BMIBC cells.

Here, we identify a KRT14‐IGF2BP1 regulatory axis that connects KRT14 function with IGF2BP1‐dependent pro‐invasive gene programs, providing a mechanistic basis for BMIBC progression and a potential therapeutic entry point.

## 2 Results

### 2.1 KRT14 Drives Invasive Progression and Lung Metastasis in BMIBC

**FIGURE 1:**
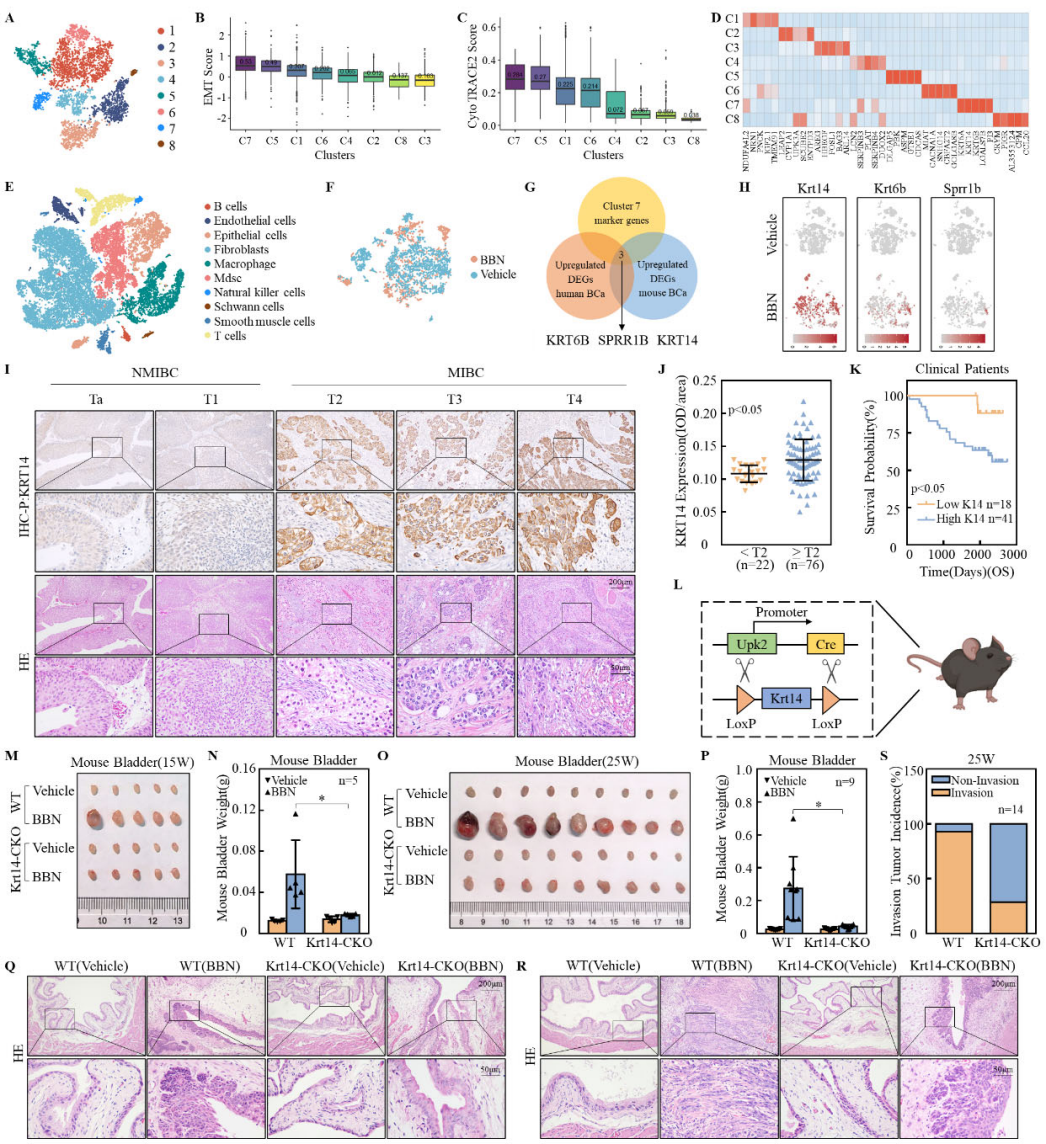
Clinical significance and functional characterization of KRT14 in BCa progression. (A) t‐SNE plot showing re‐clustered malignant epithelial cells from human BCa scRNA‐seq data (GSE267718), with distinct colors representing different cell subpopulations. (B,C) EMT and CytoTRACE2‐based stemness scores identifying subclusters with high EMT and CSC states. (D) Heatmap of representative marker genes across epithelial subclusters. (E) t‐SNE visualization of major cell populations from vehicle‐treated and BBN‐induced mouse bladders. (F) t‐SNE plot of re‐clustered epithelial subclusters. (G) Venn diagram showing overlap among the top 500 upregulated epithelial differentially expressed genes (DEGs) from human and mouse BCa and marker genes of human subcluster 7. (H) t‐SNE plots depicting expression of the three intersected genes in epithelial cells from vehicle‐treated and BBN‐treated mice. (I,J) Representative HE and IHC images and quantitative IHC scores of KRT14 in clinical BCa tissues. (K) Kaplan‐Meier overall survival curves of BCa patients stratified by KRT14 expression. (L) Schematic of the Krt14‐CKO mouse model. (M–P) Gross bladder morphology and weight comparisons in WT and Krt14‐CKO mice at 15 and 25 weeks after BBN exposure. (Q,R) Representative HE images of WT and Krt14‐CKO bladder tissues at 15 and 25 weeks after BBN exposure. (S) Quantification of bladder lesions severity in WT and Krt14‐CKO mice at 25 weeks after BBN exposure, comparing lesions with and without muscularis invasion. Data are expressed as the mean ± SD, and the symbol (*) indicates a significant difference at *p* < 0.05.

BCa comprises two major clinical subtypes: non‐muscle‐invasive bladder cancer (NMIBC) and MIBC, which differ markedly in molecular features, biological behavior, and clinical outcomes [16]. To comprehensively capture the cellular heterogeneity across these subtypes, we analyzed scRNA‐seq data from nine human BCa samples, including five MIBC and four NMIBC cases (GSE267718) [17]. After quality control, batch‐effect correction, and removal of doublets using DoubletFinder, 31 096 high‐quality cells were retained for downstream analyses. Guided by canonical marker expression and the cell‐type annotations reported in the original publication, we classified the cells into eight major types using t‐distributed stochastic neighbor embedding (t‐SNE): B cells (*CD79A*, *CD19*), endothelial cells (*PECAM1*), fibroblasts (*COL1A1*), megakaryocytes (*PF4*, *PPBP*), monocytes/macrophages (*LYZ*, *CD14*, *MRC1*, *CD68*), plasma cells (*CD79A*, *IGHG1*), T cells (*CD3D*, *CD3E*), and urothelial cells (*EPCAM*, *UPK2*, *UPK1A*) (Figure S1A,B and Table S1). A total of 5817 epithelial cells were identified based on known epithelial markers. Because the cell‐type annotation was performed according to the original publication, we hereafter use the term epithelial cells instead of urothelial cells for consistency. After reclustering and excluding contaminating immune and stromal populations, 4895 high‐confidence epithelial cells were retained for further analyses, which resolved into eight distinct subclusters (C1–C8) (Figure 1A). Cell‐origin analysis revealed that epithelial cells across all clusters were predominantly derived from MIBC samples, whereas NMIBC contributed only a small fraction (Figure S1C), suggesting that the epithelial transcriptomic landscape captured in this dataset largely reflects MIBC‐associated molecular features. Based on the expression patterns of EMT‐related genes, we calculated EMT scores for each cluster and applied CytoTRACE2 to evaluate differentiation potential and stemness. All clusters were subsequently ranked by their median scores (Figure 1B,C). Among them, Cluster 7 was distinctly characterized by prominent EMT and CSC features and exhibited high expression of KRT6A, KRT14, KRT6B, LGALS7B, and PI3 (Figure 1D). Functional enrichment analysis revealed that the top 20 marker genes of this cluster were enriched in intermediate filament‐related biological processes (Figure S1D). Among these, KRT6A, KRT14, and KRT6B, which are keratin family members and major structural components of intermediate filaments [18], ranked among the top five marker genes, suggesting their potential involvement in EMT and CSC programs characteristic of aggressive MIBC. The keratin expression pattern of Cluster 7 closely mirrors that of BMIBC, the most aggressive molecular subtype of the disease.

To further investigate the conserved epithelial programs associated with this aggressive phenotype, we employed an N‐butyl‐N‐(4‐hydroxybutyl)nitrosamine (BBN)‐induced mouse model that recapitulates key molecular and histopathological features of human BMIBC [19]. BBN, a nitrosamine alkylating compound structurally related to carcinogens found in cigarette smoke, specifically targets the bladder epithelium [20]. Continuous exposure to 0.05% BBN in drinking water for 25 weeks consistently induced invasive bladder tumors resembling human BMIBC [21] (Figure S2A). Immunohistochemical (IHC) analysis of bladder tissues collected at different stages of BBN exposure showed progressive upregulation of the canonical basal markers Krt5 and Cd44, further supporting the basal molecular identity of BBN‐induced MIBC in our model (Figure S2B–D). We then performed scRNA‐seq on bladder tissues from vehicle‐treated control mice (*n* = 2) and BBN‐induced tumor‐bearing mice (*n* = 2) at the end of the exposure period. After stringent quality filtering, 30 548 high‐quality cells were retained for downstream analysis. Dimensionality reduction and unsupervised clustering identified 25 distinct clusters (Figure S3A,B). t‐SNE visualization showed broadly comparable clustering patterns between biological replicates within each group (Figure S3C). Cell‐type composition analysis further showed that the major cell populations were represented in both replicates, supporting the reproducibility of the major cellular architecture identified by scRNA‐seq (Figure S3D). Based on canonical marker gene expression, these clusters were annotated into ten major cell types, including B cells (*Cd79a*), endothelial cells (*Cldn5, Pecam1*), epithelial cells (*Upk1b, Upk3a, Epcam*), fibroblasts (*Col6a1, Col6a2*), macrophages (*Cd68, Cd86*), mdsc (*S100a8, S100a9*), natural killer cells (*Nkg7*), schwann cells (*Sox10, Fabp7*), smooth muscle cells (*Acta2*), and T cells (*Cd3d, Cd3g*) (Figure 1E; Figure S3E). Given that epithelial cells are the direct targets of BBN‐induced transformation, we focused on this population for re‐clustering. As shown in Figure 1F, epithelial cells from vehicle‐treated and BBN‐induced mice displayed distinct distributions, highlighting tumor‐associated transcriptional changes. To ensure cross‐species comparability, a parallel analysis was performed in human BCa by integrating two scRNA‐seq datasets, including nine tumor tissues (GSE267718) and four adjacent normal bladder tissues (GSE222315) [22] (Figure S1E,F). Epithelial cells were extracted based on canonical markers (EPCAM, UPK2, UPK1A), and genes that were significantly upregulated in tumor epithelial cells relative to normal tissues were identified (Figure S1G). Subsequently, the top 500 tumor‐upregulated epithelial genes (*p* < 0.05) from both human and mouse datasets were intersected with the complete marker‐gene set of human Cluster 7, which represents the most aggressive epithelial subpopulation identified earlier. This integrative analysis revealed three conserved epithelial regulators, KRT6B, SPRR1B, and KRT14, that were consistently upregulated in both human and mouse tumor epithelia and enriched within the invasive Cluster 7 population (Figure 1G; Table S2). We next compared their epithelial expression patterns in the BBN‐induced mouse model relative to vehicle‐treated controls. Although all three genes were upregulated upon BBN exposure, Krt14 showed markedly greater induction and broader epithelial distribution than either Krt6b or Sprr1b, indicating its dominant activation during tumor progression (Figure 1H; Figure S3F). Collectively, these findings support a potential link between elevated Krt14 expression and epithelial aggressiveness in BBN‐induced tumor progression.

To validate these observations, we next examined Krt14 expression dynamics across different stages of BBN‐induced tumorigenesis in mice. Compared with vehicle‐treated controls, Krt14 mRNA levels were markedly elevated after 25 weeks of BBN exposure (Figure S4A), and its protein expression exhibited a stepwise increase at 5, 15, and 25 weeks of BBN exposure, paralleling tumor progression (Figure S4B,C). We next evaluated KRT14 expression in human BCa datasets. Analysis of the TCGA‐BLCA cohort revealed significantly higher KRT14 expression in tumors than in adjacent normal tissues (Figure S5A,B). This finding was further confirmed in the GEO dataset GSE13507 [23, 24], which also showed elevated KRT14 expression in BCa samples (Figure S5C). High‐grade MIBC specimens exhibited substantially higher KRT14 levels than low‐grade NMIBC patients (Figure S5D). Consistent with previous studies, patients with high KRT14 expression were predominantly classified into the basal subtype across all seven established MIBC classification systems [25] (Figure S5E). Moreover, KRT14 expression was significantly higher in BMIBC tissues compared with luminal MIBC (LMIBC) (Figure S5F), a result further validated in the microarray dataset GSE87304 [26] (Figure S5G). Importantly, BMIBC patients with high KRT14 expression exhibited significantly poorer survival outcomes (Figure S5H,I). Similarly, analysis of the UC‐GENOME cohort of 218 patients with metastatic urothelial carcinoma [27] showed that high KRT14 expression defined a subgroup with significantly worse survival (Figure S5J). Together, these results reinforce the prognostic significance of KRT14 in invasive urothelial carcinoma.

We then performed IHC analysis on tumor samples from 98 patients with BCa to assess KRT14 expression. IHC confirmed that KRT14 expression was markedly higher in MIBC (T stage: T2‐T4) than in NMIBC (T stage: Ta‐T1) (Figure 1I,J). Notably, a subset of MIBC cases with minimal or absent KRT14 but strong KRT20 positivity exhibited features characteristic of LMIBC [5] (Figure S5K). Consistent with transcriptomic analyses, higher KRT14 expression, as determined by IHC, was associated with significantly shorter overall survival in patients (Figure 1K). Collectively, these findings identify KRT14 as a potential biomarker of the BMIBC phenotype and suggest its possible therapeutic relevance in mitigating BCa aggressiveness.

To investigate the functional role of KRT14 in BCa progression, we generated a urothelial‐specific Krt14‐CKO mouse model using the Cre‐LoxP system [28]. In both human and mouse bladders, UPK2 is primarily expressed in intermediate and superficial epithelial cells, whereas KRT5 and KRT14 are predominantly localized in basal cells [29, 30]. As previously reported, KRT5 and KRT14 form heterodimers that are essential for maintaining epidermal structural integrity [31]. Consequently, basal layer‐specific deletion of *Krt14* using *Krt5‐*Cre would likely cause severe skin barrier defects or lethality [32, 33], precluding its application in tumorigenesis studies. To circumvent these limitations, we employed a *Upk2*‐Cre driver, which exhibits well‐validated urothelial‐specific recombination [34]. We crossed *Upk2*‐Cre mice with *Krt14* ^lox/lox^ mice to generate Krt14‐CKO offspring (Figure 1L; Figure S6A). Although Upk2 is a marker of terminal differentiation, its expression in mouse bladder development initiates in the basal layer [35]. As a result, only a portion of basal cells undergoes recombination, leading to mosaic “Krt14‐low” tumors. Validation of Krt14 knockout efficiency at both the mRNA and protein levels in age‐matched Krt14‐CKO and WT mice (Figure S6B,C) confirmed a significant, albeit incomplete, reduction of Krt14. This model effectively recapitulated the attenuated expression of Krt14 in the bladder, providing a reliable platform to assess its functional impact on BCa progression. Histopathological examination of bladder epithelium revealed no evident morphological abnormalities in Krt14‐CKO bladders (Figure S6D). We next exposed both Krt14‐CKO and WT mice to long‐term BBN treatment for 15 and 25 weeks to induce bladder tumorigenesis and progression toward BMIBC. In the vehicle group, bladder weights did not differ significantly between WT and Krt14‐CKO mice, indicating that Krt14 loss alone does not affect bladder morphology under physiological conditions. In contrast, following BBN exposure, WT mice developed markedly heavier bladder tumors than Krt14‐CKO mice, indicating that Krt14 deletion substantially attenuated tumor progression (Figure 1M–P). Histopathological evaluation of panoramic low‐magnification HE images revealed less pronounced neoplastic changes at 15 weeks (Figure 1Q; Figure S7A) and reduced muscle‐layer invasion at 25 weeks in Krt14‐CKO bladders compared with WT bladders (Figure 1R,S; Figure S8A,B). Krt14 IHC further confirmed efficient depletion of Krt14^+^ epithelial areas in Krt14‐CKO bladders (Figure S6E–G). Together, these findings suggest that Krt14 loss suppresses early neoplastic development and subsequent progression toward BBN‐induced BMIBC.

**FIGURE 2:**
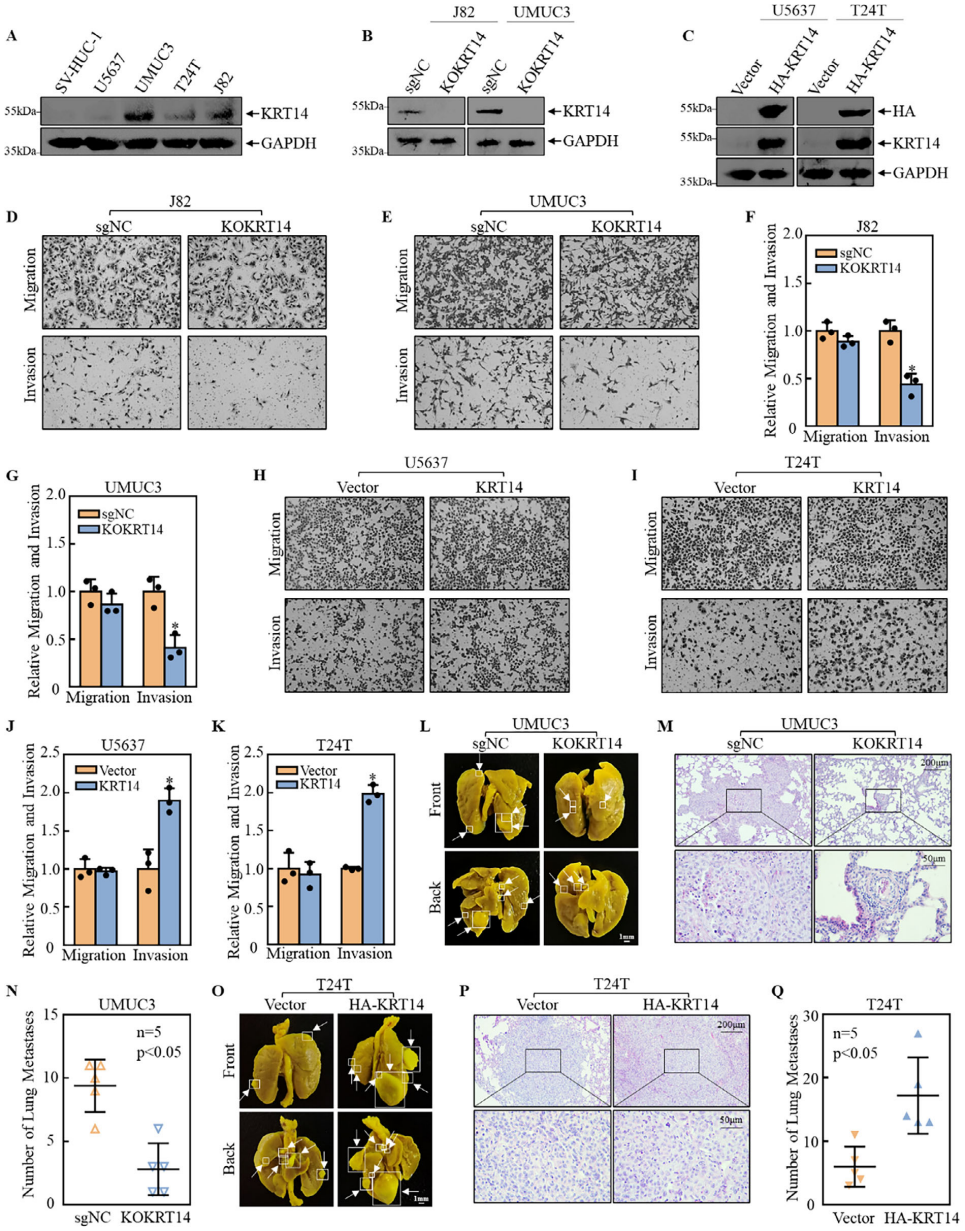
Impact of KRT14 on invasion and lung metastasis of BMIBC in vitro and in vivo. (A) Western blot analysis of KRT14 protein levels in BMIBC cell lines (U5637, UMUC3, T24T, and J82) compared with normal human urothelial cells (SV‐HUC‐1). (B) Western blot validation of KRT14 knockout generated by CRISPR‐Cas9 in J82 and UMUC3 cells. (C) Western blot validation of stable KRT14 overexpression in U5637 and T24T cells. (D–G) Representative images and quantification of migration and invasion assays in KRT14‐knockout J82 and UMUC3 cells. (H–K) Representative images and quantification of migration and invasion assays in KRT14‐overexpressing U5637 and T24T cells. (L–N) Representative lung images (front and back views), quantification of metastatic colonization, and HE staining of lung tissues from nude mice injected with KRT14‐knockout UMUC3 cells or control cells. (O–Q) Representative lung images (front and back views), quantification of metastatic colonization, and HE staining of lung tissues from nude mice injected with KRT14‐overexpressing T24T cells or control cells. Data are expressed as the mean ± SD. The symbol (*) indicates a statistically significant increase or decrease in the invasive capacity of KRT14‐knockout or KRT14‐overexpressing cells compared with their corresponding controls (*p* < 0.05).

To corroborate these findings, we conducted in vitro and in vivo assays using BMIBC cell lines. KRT14 protein levels were elevated to varying degrees in BMIBC cells compared with normal urothelial cells (SV‐HUC‐1) (Figure 2A). KRT14‐knockout cell lines were generated using CRISPR‐Cas9 in J82 and UMUC3 cells (high KRT14 expression), while KRT14‐overexpressing cell lines were established in U5637 and T24T cells (low KRT14 expression). The efficiency of KRT14 knockout and overexpression was confirmed by Western blot (Figure 2B,C). Transwell assays revealed that KRT14 knockout selectively impaired cell invasion without affecting migration (Figure 2D–G), whereas enforced KRT14 expression markedly enhanced invasion in vitro (Figure 2H–K). Correspondingly, in a lung metastasis model, KRT14 knockout significantly reduced pulmonary colonization by BMIBC cells (Figure 2L–N), while KRT14 overexpression promoted this process in vivo (Figure 2O–Q). Together, these results indicate that KRT14 contributes to invasion‐associated phenotypes and pulmonary colonization in BMIBC models, providing a mechanistic basis for further investigation.

### 2.2 IGF2BP1 Upregulation Supports KRT14‐Driven Invasion and BMIBC Progression

**FIGURE 3:**
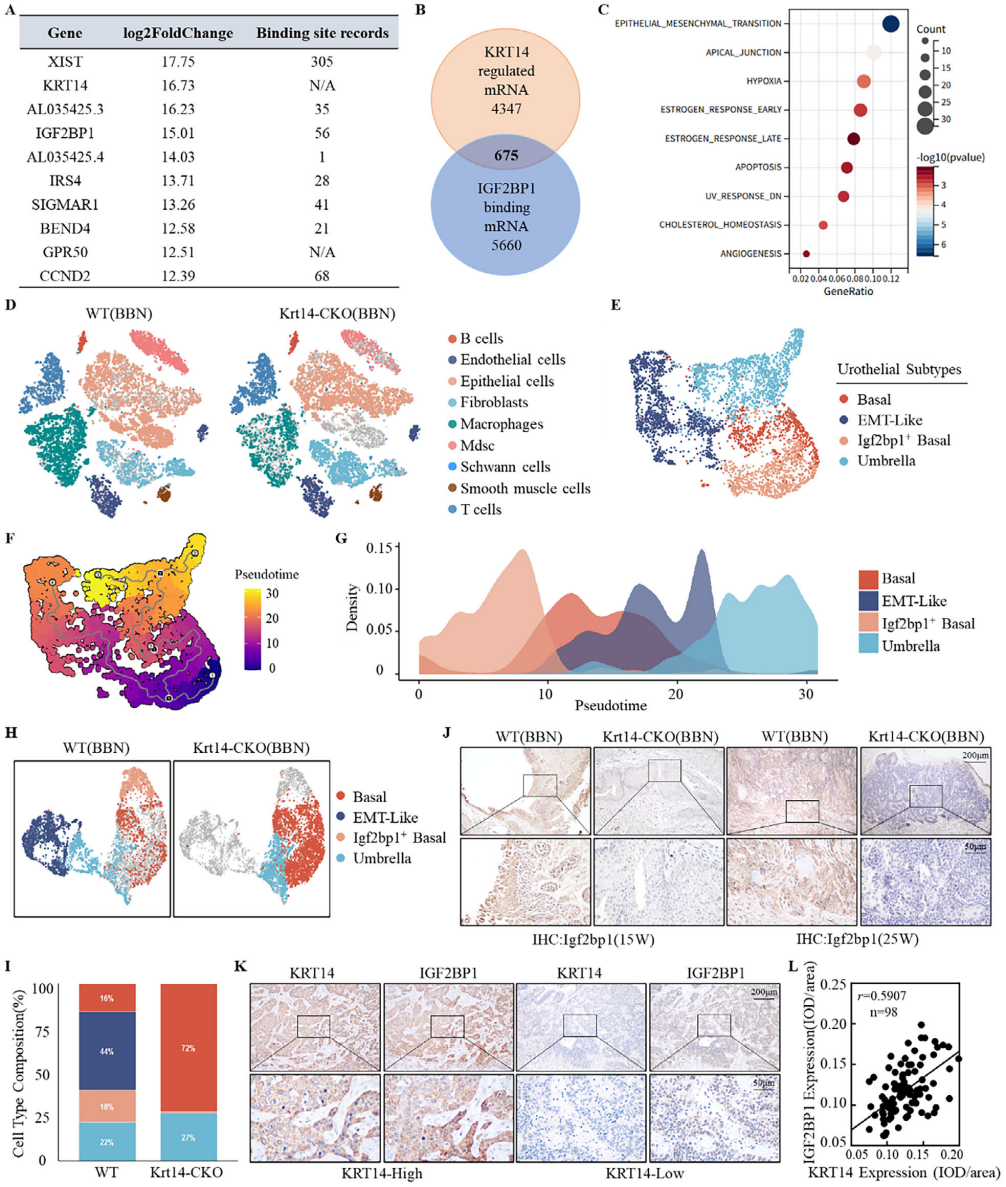
The effect of KRT14‐mediated IGF2BP1 regulation on BMIBC. (A) RNA‐seq analysis of KRT14‐overexpressing U5637 cells showing the top 10 upregulated DEGs relative to control cells. Log_2_Foldchange represents the magnitude of differential expression. Binding site records indicate the number of experimentally reported IGF2BP1‐RNA binding events for each gene, as curated from POSTAR3 database. N/A indicates that no IGF2BP1‐RNA binding events were reported for that gene. (B) Venn analysis showing the overlap between KRT14‐modulated DEGs (|log_2_FC| > 2 and padj < 0.01) and IGF2BP1‐bound transcripts retrieved from the POSTAR3 database. (C) Pathway enrichment analysis of the 675 overlapping transcripts identified in (B). (D) t‐SNE visualization of major cell types in BBN‐induced BCa tissues from WT and Krt14‐CKO mice. (E) UMAP visualization of re‐clustered urothelial cells from BBN‐induced bladder tumors in WT mice at 25 weeks post‐exposure, annotated into distinct urothelial subtypes. (F) Pseudotime trajectory analysis of the re‐clustered urothelial cells shown in (E), generated using Monocle3. (G) Pseudotime ordering of urothelial subpopulations highlighting differentiation dynamics. (H,I) t‐SNE visualization and proportional analysis of *Igf2bp1^+^* basal and EMT‐like populations in WT and Krt14‐CKO tumors. (J) IHC analysis of Igf2bp1 expression in mouse bladder epithelium from WT and Krt14‐CKO mice after BBN induction. (K,L) IHC staining of IGF2BP1 in human BCa tissues with different KRT14 levels and correlation analysis of their protein expression.

To systematically investigate the molecular alterations associated with KRT14 upregulation in BMIBC, we performed bulk RNA‐seq on KRT14‐overexpressing U5637 cells and vector controls. Notably, among the top 10 upregulated genes following KRT14 induction, 8 were previously annotated as IGF2BP1 targets in the POSTAR3 database [36] (Figure 3A), suggesting that IGF2BP1 may serve as a potential downstream mediator of KRT14‐driven transcriptional programs. IGF2BP1, a highly conserved RNA‐binding protein, plays a pivotal role in post‐transcriptional regulation by recognizing and binding to the 3'UTRs of target mRNAs, thereby stabilizing them and promoting tumor progression processes such as proliferation, invasion, and metastasis [37]. Integrative analysis identified 675 overlapping genes between KRT14‐induced differentially expressed genes (DEGs) (|log_2_FoldChange| > 2, padj < 0.01) and known IGF2BP1 targets (Figure 3B). Functional enrichment analysis of the co‐regulated genes revealed prominent enrichment in hallmark gene sets, most notably EMT and apical junction (Figure 3C). This transcriptomic landscape suggests a coordinated shift toward mesenchymal and invasion‐associated programs. These programs are associated with loss of apical‐basal polarity and acquisition of invasive traits, consistent with the pro‐invasive phenotype observed upon KRT14 overexpression [38, 39, 40]. Specifically, this enrichment involved genes related to extracellular matrix (ECM) remodeling and EMT‐associated transcriptional regulation. We selected VCAN, an invasion‐associated extracellular matrix component [41, 42], and ZEB1, a key EMT transcriptional regulator [43], to represent these two functional programs for downstream validation of the KRT14‐IGF2BP1 axis. In line with these observations, we next examined IGF2BP1 expression under KRT14 modulation. Quantitative PCR (qPCR) analysis showed that IGF2BP1 mRNA levels were significantly increased by KRT14 overexpression and reduced upon KRT14 knockout (Figure S9A–C). To determine whether these transcriptional changes were reflected at the protein level, Western blot analysis was performed and yielded consistent results (Figure S9D–F). We further examined Igf2bp1 expression in bladder tissues collected from mice at different stages of BBN‐induced tumorigenesis. Igf2bp1 expression progressively increased with tumor progression (Figure S9G,H) and displayed a strong positive correlation with Krt14 expression (Figure S9I).

Having established the association between KRT14 upregulation, IGF2BP1 expression, and EMT‐associated programs in vitro, we next examined whether Krt14 loss alters epithelial cell states during BBN‐induced bladder tumor progression in vivo by performing scRNA‐seq on Krt14‐CKO and WT bladder tissues after 25 weeks of BBN induction, together with age‐matched vehicle‐treated controls. Dimensionality reduction and unsupervised clustering identified nine major cell types based on canonical marker expression, using the same marker sets as in our previous mouse scRNA‐seq analysis to ensure consistent cell‐type annotation. Analysis of scRNA‐seq profiles was performed to compare the cellular composition between Krt14‐CKO and WT mice. At homeostasis (in the absence of BBN), the Krt14‐CKO bladder exhibited a mild shift in cellular composition, with a modest increase in fibroblast abundance and a corresponding reduction in epithelial cells, whereas other cell populations remained largely unchanged (Figure S10A). Histology confirmed normal epithelial layering and tissue morphology (Figure 1R). Upon BBN exposure, this compositional shift became more pronounced, notably marked by the loss of a specific epithelial subcluster in Krt14‐CKO tumors that was prominently represented in WT tumors (Figure 3D; Figure S10A). These findings indicate that Krt14 loss is associated with modest remodeling of the urothelial‐stromal cellular landscape, providing a cellular context that may help explain the attenuated tumor progression observed in these mice. Guided by this landscape, we focused on epithelial tumor cells to investigate how Krt14 loss impacts tumor‐associated epithelial states and transcriptional programs during bladder tumor progression.

Focusing specifically on bladder epithelial cells from BBN‐induced WT mice (*n* = 3), we re‐clustered this population and identified four distinct urothelial subtypes: (1) Basal (*Krt14^+^*), (2) EMT‐like (*Vim^+^*), (3) *Igf2bp1^+^* Basal (*Krt14^+^; Igf2bp1^+^*), and (4) Umbrella (*Upk1b^+^*) (Figure 3E; Figure S10B). To infer the developmental origin of bladder epithelial hierarchy, we assessed relative differentiation potential using CytoTRACE2. While the highest scores (“totipotency”) were observed in the EMT‐like cluster, elevated pluripotency and multipotency were also present in the *Igf2bp1^+^* basal population (Figure S10C). Because high potency in tumors can reflect either progenitor identity or acquired plasticity through de‐differentiation [44], we designated the *Igf2bp1^+^* basal population as the root of the Monocle3 pseudotime trajectory, guided by the established progenitor identity of basal cells. This configuration revealed a clear progression toward the EMT‐like and other terminal clusters (Figure 3F,G; Figure S10D,E) and indicated that the peak CytoTRACE2 scores observed in EMT‐like cells are most consistent with de‐differentiation‐associated plasticity [45]. These results identify the *Igf2bp1^+^* basal cells as the developmental origin of the tri‐branched epithelial hierarchy (Figure 3F). Comparative re‐analysis of bladder urothelial cells from BBN‐induced WT and Krt14‐CKO mice revealed two major phenotypic changes in the Krt14‐CKO group, characterized by a near‐complete loss of the *Igf2bp1^+^* basal subpopulation and an almost complete absence of EMT‐like cells (Figure 3H,I; Figure S10F). Collectively, these findings suggest a functional association between the KRT14‐IGF2BP1 axis and the emergence and maintenance of EMT‐like states, indicating that *Krt14^+^ Igf2bp1^+^* basal cells occupy an upstream position in the epithelial differentiation hierarchy and contribute to metastatic progression in BCa.

To validate the KRT14‐IGF2BP1 relationship at the protein level in vivo, we examined their co‐expression. In BBN‐induced Krt14‐CKO mice, Igf2bp1 protein levels were markedly reduced in parallel with Krt14 loss (Figure 3J; Figure S11A,B). Similarly, analysis of clinical BCa specimens revealed a strong positive correlation between KRT14 and IGF2BP1 protein expression (Figure 3K,L), supporting their regulatory association in human BCa. Given the correlation observed in vivo, we next tested whether IGF2BP1 functions as a critical downstream effector of KRT14‐driven invasion. IGF2BP1 was either knocked out or overexpressed in KRT14‐overexpressing and KRT14‐knockout BMIBC cells, respectively, with successful perturbations confirmed by Western blot (Figure S12A–C). In Transwell assays, IGF2BP1 knockout markedly attenuated the invasive phenotype induced by KRT14 overexpression (Figure S12D–G), whereas IGF2BP1 overexpression fully rescued the impaired invasive phenotype observed in KRT14‐knockout cells (Figure S12H,I). Taken together, these results support the existence of a KRT14‐IGF2BP1 signaling axis that sustains malignant basal and EMT‐like subpopulations and drives tumor invasion, offering mechanistic insight into BMIBC progression.

Because BMIBC often displays squamous‐associated features, we next examined this phenotype in BBN‐induced tumors after Krt14 deletion [15, 25]. At 25 weeks, histological assessment revealed areas showing morphological features of squamous differentiation in Krt14‐CKO tumors (Figure S8A,B). Based on previously reported squamous differentiation‐related gene sets, including cornification‐related genes such as Ivl and Sprr1a and desmosome‐related genes such as Dsg3 [46], scRNA‐seq analysis of tumor epithelial cells showed higher Dsg3 and Ivl expression in Krt14‐CKO tumors than in WT tumors, whereas Sprr1a showed no obvious difference between the two groups (Figure S13A–F). These results showed that histological squamous features were still observed after Krt14 deletion, with variable changes among selected squamous‐associated markers.

We further examined whether Krt14 deletion altered the immune contexture of BBN‐induced tumors. After BBN induction, Krt14‐CKO bladders showed a lower proportion of myeloid cells, including macrophages and MDSCs, and a higher proportion of T cells than WT bladders (Figure S14A). Because PD‐1/PD‐L1 blockade is widely used in BCa treatment [47], we next assessed transcriptional features associated with response to this therapy. Using scRNA‐seq profiles from WT and Krt14‐CKO bladder tumors, we calculated the IFNγ score and T cell‐inflamed gene expression profile score (GEP) described by Ayers et al. [48]. Both scores were higher in Krt14‐CKO tissues than in WT tissues (Figure S14B,C). These results suggested that Krt14 deletion was associated with a more T cell‐inflamed immune profile during BBN‐induced bladder tumor progression, accompanied by transcriptional features linked to potential responsiveness to immune checkpoint blockade. Overall, Krt14 loss was associated with both attenuated KRT14‐IGF2BP1‐related EMT and invasion programs and remodeling of the immune microenvironment during BBN‐induced bladder tumor progression.

### 2.3 KRT14 Directly Binds the IGF2BP1 KH2 Domain Through Its N‐Terminal Region

**FIGURE 4:**
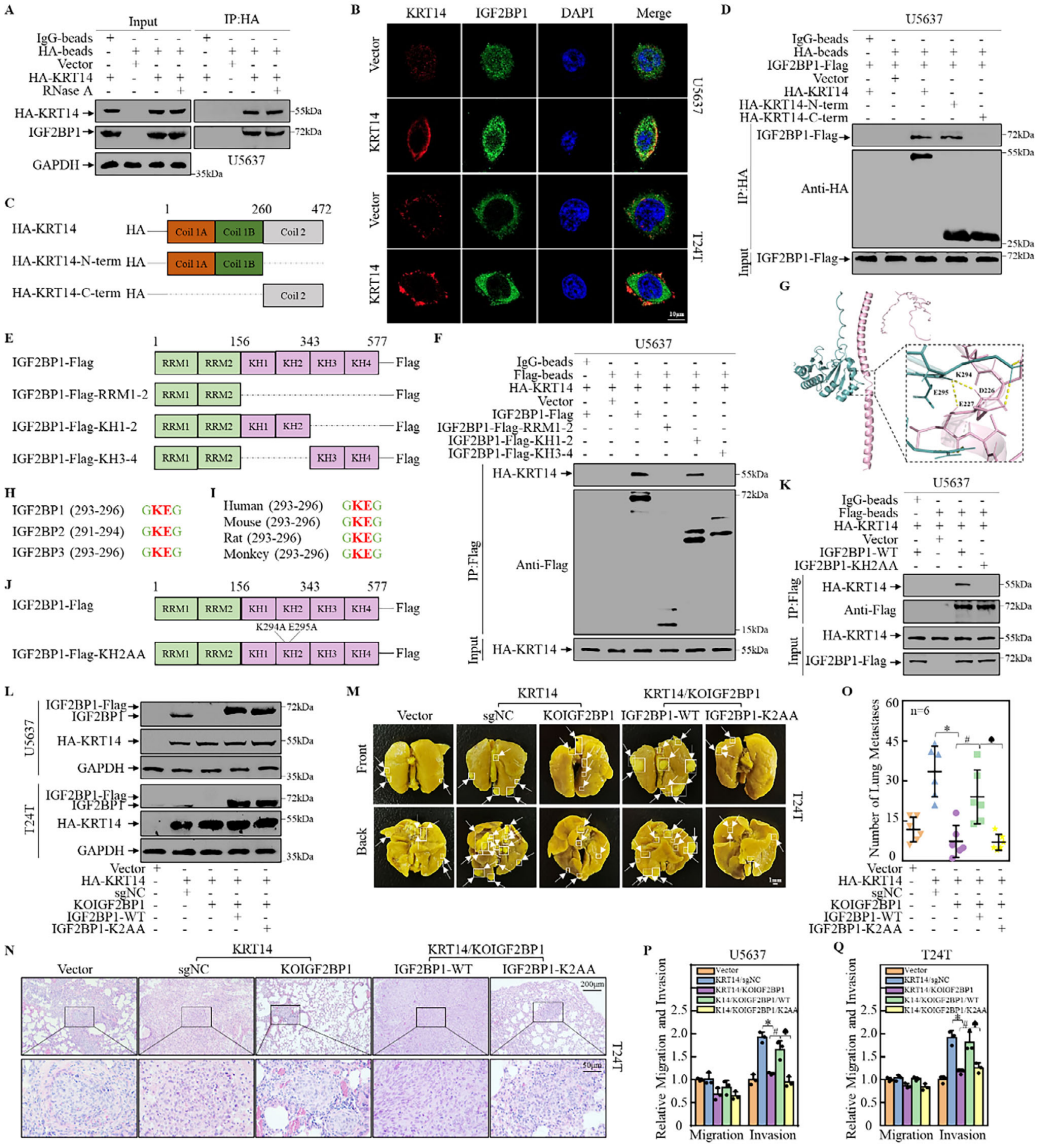
KRT14 binds IGF2BP1 and regulates tumor cell invasion and pulmonary colonization. (A) Co‐IP showing KRT14‐IGF2BP1 interaction in the presence or absence of RNase A. (B) Representative IF images showing subcellular co‐localization of KRT14 and IGF2BP1 in BMIBC cells. (C) Schematic representation of the KRT14 truncation constructs, including the N‐terminal fragment (1–260 aa) and the C‐terminal fragment (261–472 aa). (D) Co‐IP analysis mapping the KRT14 region required for interaction with IGF2BP1 using the truncation constructs shown in (C). (E) Schematic representation of the IGF2BP1 truncation constructs, including the RRM1‐2 fragment (1–156 aa), the KH1‐2 fragment (1–343 aa), and the KH3‐4 fragment (1–156 aa and 344–577aa). (F) Co‐IP analysis identifying the IGF2BP1 domain involved in its interaction with KRT14 using the truncation constructs shown in (E). (G) DMFold modeling predicting interaction between the KRT14 N‐terminal region (pink) and the IGF2BP1 KH2 domain (cyan). (H,I) Conservation of the KH2‐domain GXXG RNA‐binding motif among IGF2BP family members and across species. (J) Schematic strategy for constructing IGF2BP1 KH2‐domain mutants with point substitutions (K294A and E295A). (K) Co‐IP analysis validating the contribution of the KH2 domain to the IGF2BP1‐KRT14 interaction using the mutants generated in (J). (L) Western blot analysis of IGF2BP1 expression in KRT14‐overexpressing BMIBC cells following IGF2BP1 knockout and rescue with IGF2BP1‐WT or IGF2BP1‐K2AA. (M–O) Representative lung images (front and back views), quantification of metastatic colonization, and HE staining of lung tissues from nude mice injected with KRT14‐overexpressing T24T cells after IGF2BP1 knockout and rescue with IGF2BP1‐WT or IGF2BP1‐K2AA. (P,Q) Quantification of migration and invasion assays in KRT14‐overexpressing U5637 and T24T cells subjected to IGF2BP1 knockout and rescue with IGF2BP1‐WT or IGF2BP1‐K2AA. Data are expressed as the mean ± SD. The symbol (*) indicates a significant reduction in invasive and metastatic abilities in KRT14‐overexpressing cells after IGF2BP1 knockout compared with control cells (*p* < 0.05). The symbol (#) indicates a significant restoration after IGF2BP1‐WT rescue compared with IGF2BP1‐knockout cells (*p* < 0.05). The symbol (♠) indicates a significant reduction after IGF2BP1‐K2AA rescue compared with IGF2BP1‐WT rescue (*p* < 0.05).

To investigate the mechanistic relationship between KRT14 and IGF2BP1, we first performed co‐immunoprecipitation (Co‐IP) assays in KRT14‐overexpressing cells. IGF2BP1 was detected in HA‐KRT14‐immunoprecipitated complexes, indicating a physical association between the two proteins (Figure S15A). To further substantiate the physiological relevance of this interaction, endogenous reciprocal Co‐IP using native KRT14 and IGF2BP1 antibodies supported the association between KRT14 and IGF2BP1 in BMIBC cells, with no specific signal detected in the isotype‐matched IgG control lanes (Figure S15B,C). Treatment with RNase A did not disrupt this interaction, confirming that KRT14 and IGF2BP1 bind in an RNA‐independent manner (Figure 4A). To further confirm their association, immunofluorescence (IF) analysis was performed to define their subcellular localization. KRT14 was primarily localized at the cell membrane, whereas IGF2BP1 exhibited a cytoplasmic distribution. Marked co‐localization was observed at the cytoplasmic periphery, suggesting a functional interplay at the membrane‐cytoplasm interface (Figure 4B). To precisely map their interaction regions, a series of domain‐deletion mutants was constructed for both proteins. KRT14 was truncated into an N‐terminal fragment (amino acids, aa 1–260) and a C‐terminal fragment (261–472 aa) (Figure 4C), whereas IGF2BP1 was divided into RRM1‐2 domains (1–156 aa) and KH1‐4 domains (157–577 aa) [49, 50] (Figure S15D). Co‐IP assays demonstrated that the N‐terminal region of KRT14 specifically interacted with the KH1‐4 domain of IGF2BP1 (Figure 4D; Figure S15E). Further subdivision of the KH1‐4 domain into KH1‐2 (1–343 aa) and KH3‐4 (1–156 aa + 344–577 aa) fragments [49, 50] (Figure 4E) revealed that deletion of KH1‐2 markedly weakened the interaction, indicating that this region is critical for binding (Figure 4F).

To further elucidate the structural basis of the KRT14‐IGF2BP1 interaction, we performed DMFold‐based structural modeling to predict potential binding interfaces between the KRT14 N‐terminus and the KH1‐2 domains of IGF2BP1 [51]. The models converged on a binding conformation in which KH2, but not KH1, established stable contacts with KRT14 (Figure 4G; Figure S15F). Molecular visualization in PyMOL, using default hydrogen‐bond assignment parameters, revealed putative hydrogen bonds at the interface. In this model, the IGF2BP1 GXXG loop (293–296 aa) interacts with a putative LXXL‐containing nuclear export sequence (NES) within KRT14 (216–230 aa) [52, 53]. Although the NES of KRT14 has not been experimentally characterized, analysis using the LocNES algorithm identified a high‐confidence NES motif in this region (score = 0.660) (Figure S15G), which closely resembles the validated NES of KRT17 [52, 54]. This finding provides independent support for the predicted interaction interface between IGF2BP1 and KRT14, suggesting that the GXXG motif contributes to protein‐protein recognition in addition to its canonical role in RNA binding. Comparative sequence analysis showed that this motif is highly conserved across species and among IGF2BP family members, underscoring its structural and functional importance (Figure 4H,I). To experimentally validate this structural prediction, we introduced a GAAG point mutation into the GKEG motif within the KH2 domain [55] (Figure 4J). Consistent with the DMFold simulation predicting that the KH2 (GAAG) mutant fails to form stable contacts with the KRT14 N‐terminus (Figure S15H), this mutation completely abolished formation of the KRT14‐IGF2BP1 complex in Co‐IP assays (Figure 4K), demonstrating that the GKEG motif is indispensable for their interaction.

To validate the functional significance of this structural interaction, IGF2BP1 was knocked out in KRT14‐overexpressing cells, which were then reconstituted with either wild‐type IGF2BP1 (IGF2BP1‐WT) or the KH2‐domain mutant (IGF2BP1‐KH2AA) (Figure 4L). Compared with IGF2BP1‐WT, IGF2BP1‐KH2AA substantially attenuated KRT14‐driven pulmonary metastatic colonization in vivo (Figure 4M–O) and reduced BMIBC cell invasion in vitro (Figure 4P,Q; Figure S16A,B). Consistently, KRT14‐knockout cells exhibited a similar pattern of phenotypic modulation in the same complementation assay (Figure S16C), further supporting the functional coupling between KRT14 and IGF2BP1 in regulating invasion‐associated phenotypes (Figure S16D,E). Together, these findings define a structure–function mechanism by which the N‐terminal region of KRT14 engages the KH2 domain of IGF2BP1 to sustain IGF2BP1‐dependent invasion and pulmonary metastatic colonization in BMIBC models.

### 2.4 KRT14 D226 and E227 Enable KRT14‐IGF2BP1 Binding and Auto‐Stabilization of IGF2BP1 mRNA

**FIGURE 5:**
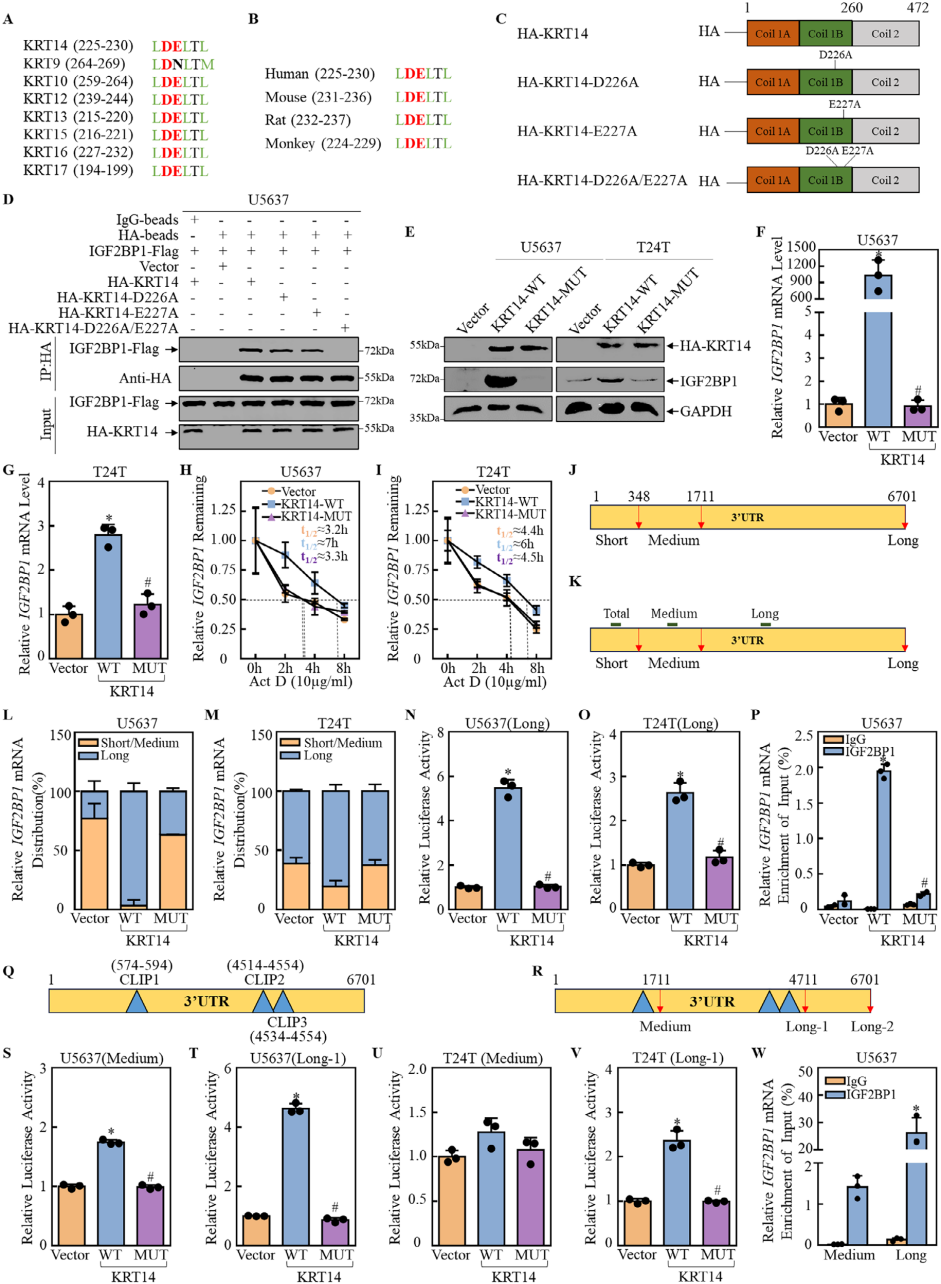
Mechanism of KRT14‐mediated enhancement of IGF2BP1's self‐mRNA recognition. (A,B) Sequence alignment of the KRT14 putative NES motif among type I keratins and across mammalian species. (C) Schematic of KRT14 mutants disrupting the predicted IGF2BP1‐binding site. (D) Co‐IP analysis identifying key residues in KRT14 required for binding IGF2BP1. (E–G) Western blot and qPCR analyses examining the effects of KRT14‐WT and KRT14‐MUT on IGF2BP1 protein and mRNA expression in U5637 and T24T cells. (H,I) Evaluation of IGF2BP1 mRNA stability in BMIBC cells expressing KRT14‐WT or KRT14‐MUT following treatment with Act D (10 µg/mL) for 2, 4, and 8 h. (J) Schematic illustration of IGF2BP1 3'UTR truncation variants generated from the 3'UTR start site. (K) qPCR primer pairs were designed in short, medium, and long regions of the IGF2BP1 3'UTR to specifically detect full‐length, medium‐length, and long‐length variants, respectively. (L,M) Relative abundance of Short/Medium versus Long IGF2BP1 3'UTR isoforms in KRT14‐WT and KRT14‐MUT BMIBC cells. (N,O) Luciferase reporter activity assays analyzing the regulatory effects of KRT14‐WT or KRT14‐MUT on the full‐length IGF2BP1 3'UTR. (P) RIP analysis assessing the interaction between IGF2BP1 and its own mRNA in KRT14‐WT and KRT14‐MUT cells. (Q) PAR‐CLIP experimental data presented in POSTAR3 showing IGF2BP1‐binding elements within its 3'UTR in HEK293T cells. (R) Construction of dual‐luciferase reporter plasmids containing progressively truncated IGF2BP1 3'UTR fragments based on the PAR‐CLIP‐identified binding regions, including the medium fragment (1–1711 nt), Long‐1 fragment (1712–4711 nt), and Long‐2 fragment (4712–6701 nt). (S–V) Dual‐luciferase reporter activity experiments examining the effects of KRT14‐WT and KRT14‐MUT cells on the reporter activity of the Medium and Long‐1 IGF2BP1 3'UTR fragments. (W) RIP assays showing that IGF2BP1 binds to regions within its 3'UTR, where CLIP1 is covered by the Medium primer set, and CLIP2/CLIP3 are covered by the Long primer set. Data are expressed as the mean ± SD. The symbol (*) indicates statistically significant increases in IGF2BP1 mRNA levels, 3′UTR reporter activity, and self‐mRNA binding, as well as stronger IGF2BP1 binding to CLIP2/CLIP3 compared with CLIP1 (*p* < 0.05). The symbol (#) indicates significantly lower IGF2BP1 mRNA levels, 3′UTR reporter activity, and self‐mRNA binding in KRT14‐MUT cells compared with KRT14‐WT (*p* < 0.05).

Building upon our previous findings that KRT14 directly modulates IGF2BP1 function through specific interactions between its N‐terminal region and the KH2 domain of IGF2BP1, we next sought to identify the key residues mediating this interaction. Molecular modeling revealed that residues K294 and E295 in the KH2 domain form stable hydrogen bonds with D226 and E227 of KRT14, two acidic residues located immediately adjacent to the LXXL core of its putative NES motif. Comparative sequence analysis further demonstrated that these interacting residues represent defining molecular features of type I keratins and are highly conserved across mammalian species (Figure 5A,B), suggesting a conserved molecular interface potentially critical for BMIBC progression. Notably, structural modeling indicated that the GXXG motif of IGF2BP1 acts as a structural probe fitting into the charged groove created by the predicted NES of KRT14, forming a ‘lock‐and‐key’ configuration that supports highly specific molecular recognition between the two proteins.

To functionally validate these structural observations, we generated alanine‐substituted variants of KRT14, including single mutants (D226A and E227A) and a double mutant (D226A/E227A, hereafter referred to as KRT14‐MUT) (Figure 5C). Co‐IP assays revealed that single‐point substitutions partially reduced IGF2BP1 binding, whereas the double mutation (KRT14‐MUT) almost completely abolished the interaction (Figure 5D; Figure S15I). These results indicate that residues D226 and E227 of KRT14 act cooperatively and are essential for binding to K294 and E295 of IGF2BP1, consistent with the hydrogen‐bonding interactions predicted by structural modeling. Having established the importance of D226 and E227 in mediating KRT14‐IGF2BP1 binding, we next investigated how this interaction regulates IGF2BP1 expression. KRT14‐MUT cells displayed markedly reduced IGF2BP1 protein levels compared with KRT14‐WT cells (Figure 5E). To determine whether this reduction occurred at the transcript level, we assessed IGF2BP1 mRNA abundance and stability (Figure 5F–I), both of which were increased in KRT14‐WT but markedly decreased in KRT14‐MUT cells. In contrast, IGF2BP1 promoter activity showed an opposite trend, being elevated in KRT14‐MUT cells but suppressed in WT cells (Figure S17A,B), indicating that the reduction of IGF2BP1 in the mutant is not transcriptionally regulated. Together, these findings suggest that KRT14 enhances IGF2BP1 protein expression primarily by stabilizing IGF2BP1 mRNA, a process dependent on the D226 and E227 residues of KRT14.

The 3'UTR plays a crucial role in post‐transcriptional regulation by modulating mRNA stability, translation efficiency, and subcellular localization [56]. IGF2BP1 is transcribed into three variants differing in 3'UTR lengths: 348 nt (Short), 1711 nt (Medium), and 6701 nt (Long) [57] (Figure 5J; Figure S17C). Previous studies have shown that alternative polyadenylation (APA) regulates IGF2BP1 3'UTR length, where shortening of the long isoform eliminates *let‐7* miRNA‐mediated repression, thereby enhancing IGF2BP1 expression in aggressive cancers [58, 59]. However, more recent evidence indicates that the long transcript variant predominates in tumor tissues and substantially contributes to IGF2BP1 protein abundance, suggesting that its post‐transcriptional upregulation mainly occurs through APA‐independent mechanisms, possibly involving reduced *let‐7* activity [57]. The positions of qPCR primer pairs targeting the Long and Medium 3'UTR variants, along with an additional primer pair detecting total IGF2BP1 mRNA, are shown in Figure 5K. Using these variant‐specific primers, we found that KRT14‐WT overexpression markedly increased the proportion of long IGF2BP1 transcripts, whereas this effect was completely lost in KRT14‐MUT cells (Figure 5L,M). Consistently, KRT14‐WT significantly enhanced the luciferase reporter activity of the longest IGF2BP1 3'UTR constructs, while KRT14‐MUT had no detectable effect (Figure 5N,O). Notably, KRT14 overexpression did not induce consistent changes in *let‐7* expression across cell lines. In U5637 cells, *let‐7* levels remained largely unchanged, whereas in T24T cells, KRT14 overexpression markedly increased *let‐7* expression (Figure S17D,E). These results suggest that KRT14‐mediated stabilization of IGF2BP1 mRNA is not primarily driven by let‐7 modulation.

Recent studies have revealed that IGF2BP1 establishes a self‐reinforcing autoregulatory loop by stabilizing its own mRNA in ovarian cancer [57]. To determine whether this mechanism is conserved in BCa and to provide a basis for evaluating its potential regulation by KRT14, we transfected U5637 and T24T cells with increasing amounts of IGF2BP1‐Flag plasmid. Western blot analysis showed a pronounced dose‐dependent increase in total IGF2BP1 protein levels (Figure S18A). Consistently, qPCR using primers targeting the 3'UTR regions of IGF2BP1 mRNA revealed that ectopic IGF2BP1 expression dose‐dependently elevated endogenous IGF2BP1 transcript abundance (Figure S18B,C), prolonged the half‐life of the endogenous mRNA (Figure S18D,E), and enhanced IGF2BP1 3'UTR luciferase reporter activity (Figure S18F,G), thus confirming the existence of a self‐reinforcing regulatory circuit in BCa cells. This positive feedback effect was more pronounced in T24T cells (with high basal IGF2BP1 expression) than in U5637 cells (with low basal levels) but plateaued beyond a critical threshold, suggesting an intrinsic homeostatic mechanism constraining the autoregulatory loop. Subsequently, we generated RNA‐binding motif mutants in each of the KH domains of IGF2BP1 to determine which domain mediates IGF2BP1‐driven mRNA stabilization [60]. Given that KH domains typically function in tandem, we generated paired mutants targeting KH1‐2 and KH3‐4. The canonical RNA‐binding motifs “GKEG” in KH1‐2 were substituted with “GDDG,” whereas “GKKG” and “GKGG” in KH3‐4 were replaced with “GEEG,” introducing negatively charged residues to disrupt RNA binding (Figure S18H). In contrast, for KRT14 interaction assays, the “GKEG” motif in the KH2 domain was replaced with “GAAG” solely for structural mapping, as this conservative substitution preserves RNA‐binding capability. These charge‐reversal mutations abolish RNA binding by KH domains without affecting domain stability [55]. To exclude expression bias, we confirmed that both mutants were expressed at levels comparable to IGF2BP1‐WT, ensuring that subsequent analyses reflected functional rather than expression differences (Figure S18I). RNA Immunoprecipitation (RIP) assays indicated that all four KH domains (KH1‐4) contribute to IGF2BP1 mRNA stabilization, with disruption of the KH3‐4 tandem markedly impairing mRNA association, whereas mutation of KH1‐2 caused only a minor reduction (Figure S18J). These findings identify the KH3‐4 tandem as the principal functional module mediating IGF2BP1 autoregulatory stabilization of its own mRNA and suggest that the KRT14‐IGF2BP1 interaction exerts only a limited influence on this self‐stabilizing mechanism.

To determine whether KRT14 regulates IGF2BP1 mRNA stability by modulating the interaction between IGF2BP1 and its own transcript, we performed RIP assays, revealing that KRT14‐WT, but not KRT14‐MUT, significantly enhanced IGF2BP1 binding to its own mRNA (Figure 5P). To further identify the regions by which KRT14 influences IGF2BP1‐mRNA interaction, we referred to previously reported PAR‐CLIP data. Using this high‐resolution method for mapping RNA‐protein interactions, Markus Hafner and colleagues identified three sites within the IGF2BP1 3'UTR that directly bind IGF2BP1 in HEK293 cells: CLIP1 (574–594 nt), CLIP2 (4514–4554 nt), and CLIP3 (4534–4554 nt) [36] (Figure 5Q). We therefore constructed three IGF2BP1 3'UTR truncation plasmids to determine which regions mediate KRT14‐mediated regulation of IGF2BP1 mRNA stability. The construct containing the CLIP1 site was designated as Medium (1–1711 nt), the fragment encompassing both CLIP2 and CLIP3 sites as Long‐1 (1712–4711 nt), and the downstream region lacking any CLIP sites as Long‐2 (4712–6701 nt) (Figure 5R). 3'UTR luciferase reporter assays revealed a distinct pattern of KRT14‐dependent regulation among these fragments. In both BMIBC cell lines, KRT14‐WT markedly increased luciferase reporter activity of the Long‐1 fragment containing the CLIP2/CLIP3 sites, whereas Long‐2 fragment lacking these sites showed no significant change. The Medium fragment, which includes the CLIP1 site, exhibited a moderate but detectable increase in U5637 cells (Figure 5S–V; Figure S17F,G), suggesting that KRT14 may also act through CLIP1 under conditions of low endogenous IGF2BP1 expression. In contrast, KRT14‐MUT failed to induce any change in luciferase reporter activity, confirming the requirement of residues D226 and E227 for this regulatory interaction. Notably, the qPCR primer pairs designed in Figure 5K amplify regions encompassing CLIP1 (Medium) and CLIP2/CLIP3 (Long‐1), allowing direct detection of IGF2BP1‐bound regions within its own 3'UTR. Consistent with this design, RIP assays showed that IGF2BP1 bound the Long fragment much more strongly than the Medium fragment, indicating preferential interaction via CLIP2/CLIP3 regions (Figure 5W). Together, these findings demonstrate that KRT14‐mediated stabilization of IGF2BP1 mRNA primarily occurs through binding to the CLIP2/CLIP3 elements within its 3'UTR.

### 2.5 KRT14 Coordinates IGF2BP1 mRNA Localization and Pro‐Invasive Cargo Transcript Regulation to Sustain Invasive Programs in BMIBC

**FIGURE 6:**
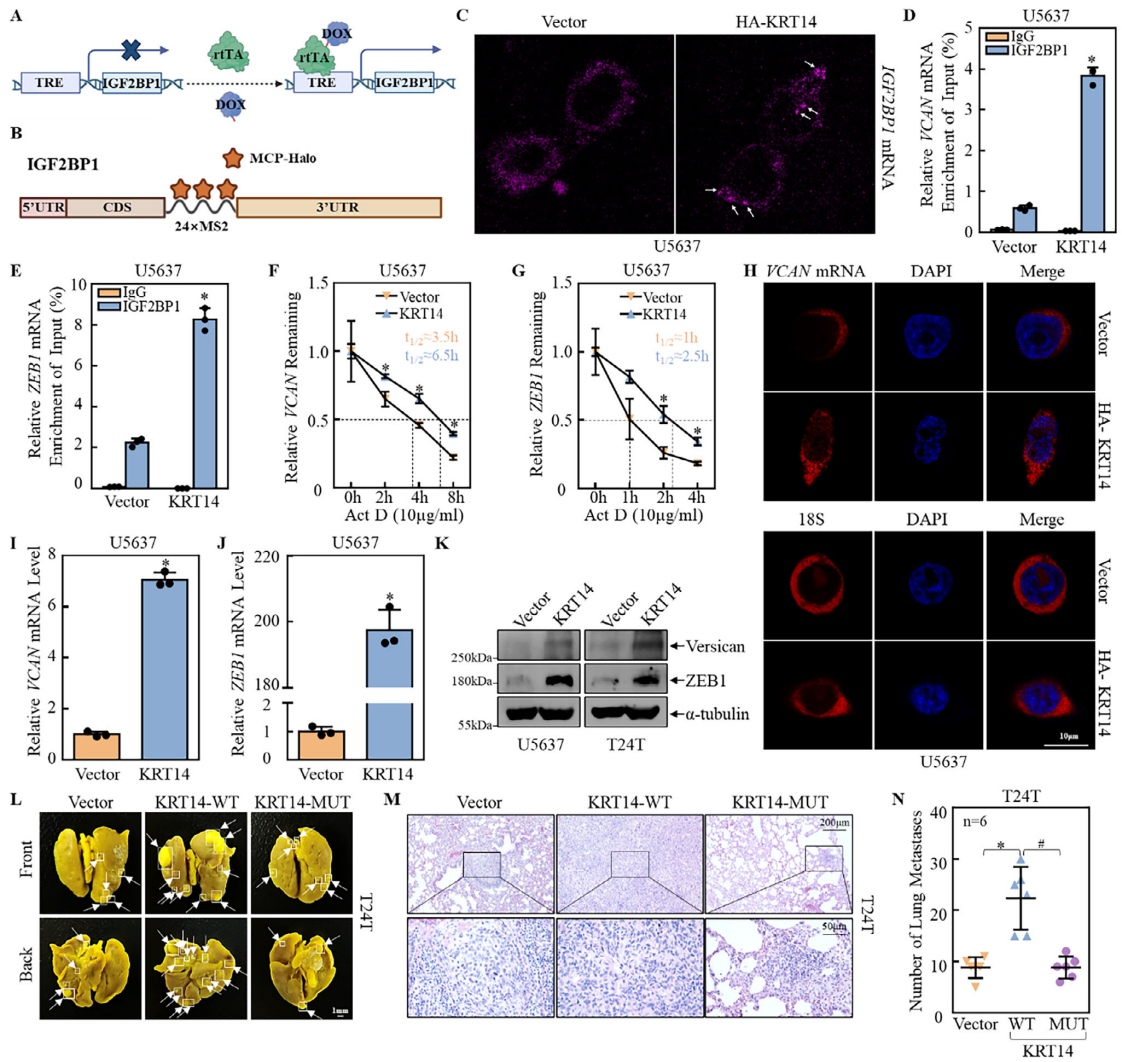
KRT14 regulates IGF2BP1 mRNA localization and pro‐invasive cargo transcript programs in BMIBC cells. (A) Schematic of the Tet‐On system in which full‐length IGF2BP1 is placed under the control of the TRE promoter module and induced by doxycycline (Dox) through rtTA activation. (B) Design of IGF2BP1 reporter constructs for single‐molecule mRNA imaging using the MS2‐MCP system, where 24×MS2 stem‐loop sequences were inserted between the IGF2BP1 CDS and its 3′UTR to enable specific MCP‐Halo binding and visualization. (C) Representative images showing subcellular localization of IGF2BP1 mRNA in U5637 cells expressing HA‐KRT14 or control vector. White arrows indicate IGF2BP1 mRNA‐MS2‐MCP‐Halo complexes. (D,E) RIP‐qPCR analysis showing IGF2BP1 binding to *VCAN* and *ZEB1* mRNAs in KRT14‐overexpressing and control cells. (F,G) Evaluation of *VCAN* and *ZEB1* mRNA stability in KRT14‐overexpressing U5637 cells and corresponding control cells following treatment with Act D (10 µg/mL) at the indicated time points. (H) Representative RNA‐FISH images showing subcellular localization of *VCAN* mRNA in U5637 cells expressing HA‐KRT14 or control vector. 18S rRNA was included as a control for general RNA distribution. (I–K) qPCR and Western blot analyses assessing the effects of KRT14 on VCAN and ZEB1 mRNA and protein levels in BMIBC cells. (L–N) Representative lung images (front and back views), quantification of metastatic colonization, and HE staining of lung tissues from nude mice injected with T24T cells expressing KRT14‐WT or KRT14‐MUT. Data are expressed as the mean ± SD. The symbol (*) indicates a significant increase in the indicated parameter in KRT14‐WT cells compared with control cells (*p* < 0.05). The symbol (#) indicates a significant decrease in the indicated parameter in KRT14‐MUT cells compared with KRT14‐WT cells (*p* < 0.05).

Our previous findings revealed that KRT14 physically associates with IGF2BP1 via its N‐terminal residues and facilitates the binding of IGF2BP1 to its own mRNA, thereby enhancing IGF2BP1 mRNA stability and increasing IGF2BP1 protein expression. Given the co‐localization of KRT14 and IGF2BP1 at the membrane‐cytoplasm interface (Figure 4B), we next asked whether KRT14 shapes the spatial organization of IGF2BP1‐related mRNA regulatory complexes. We hypothesized that KRT14 promotes the peripheral recruitment of IGF2BP1 mRNA and spatial regulation of IGF2BP1‐associated cargo transcripts, thereby supporting localized post‐transcriptional regulation and invasive cellular behavior. To visualize IGF2BP1 mRNA dynamics, we constructed a Tet‐on reporter containing the full‐length 5'UTR, CDS, and 3'UTR of IGF2BP1 (Figure 6A). For live‐cell visualization of individual mRNA molecules, 24xMS2 stem‐loop repeats were inserted into the 3'UTR, allowing recruitment of multiple MCP‐Halo fusion proteins for single‐molecule tracking [61, 62] (Figure 6B). This well‐established tagging strategy enables high‐resolution RNA imaging without markedly altering mRNA structure or trafficking [62]. Upon doxycycline induction, KRT14 overexpression markedly promoted the accumulation and directionally biased movement of IGF2BP1 mRNA toward the cytoplasmic periphery and protrusive regions, whereas control cells exhibited slower, less organized, and more randomly diffusive trajectories (Figure 6C; Video S1).

After establishing that KRT14 promotes peripheral localization of IGF2BP1 mRNA, we asked whether this spatial regulation also extends to IGF2BP1‐bound pro‐invasive transcripts. Based on the ECM‐remodeling and EMT‐associated signature described above, we selected VCAN and ZEB1 for validation. RIP confirmed IGF2BP1 binding to both *VCAN* and *ZEB1* mRNAs, with stronger enrichment after KRT14 overexpression (Figure 6D,E). Actinomycin D chase assays showed prolonged half‐lives of both transcripts after KRT14 overexpression, consistent with post‐transcriptional stabilization through the KRT14‐IGF2BP1 axis (Figure 6F,G; Figure S19A,B). RNA‐FISH further revealed that *VCAN* mRNA signals were more enriched at the peripheral cytoplasm in KRT14‐overexpressing cells than in vector control cells, supporting KRT14‐dependent spatial regulation of this ECM‐related cargo (Figure 6H). Consistent with these results, KRT14 overexpression increased VCAN and ZEB1 mRNA and protein levels (Figure 6I–K; Figure S19C,D). These findings indicate that KRT14 enhances IGF2BP1‐dependent binding and stabilization of pro‐invasive cargo mRNAs, including VCAN and ZEB1. In this context, VCAN further displayed redistribution toward peripheral cytoplasmic regions, linking KRT14‐IGF2BP1‐dependent cargo regulation to ECM‐remodeling and EMT‐associated programs.

**FIGURE 7:**
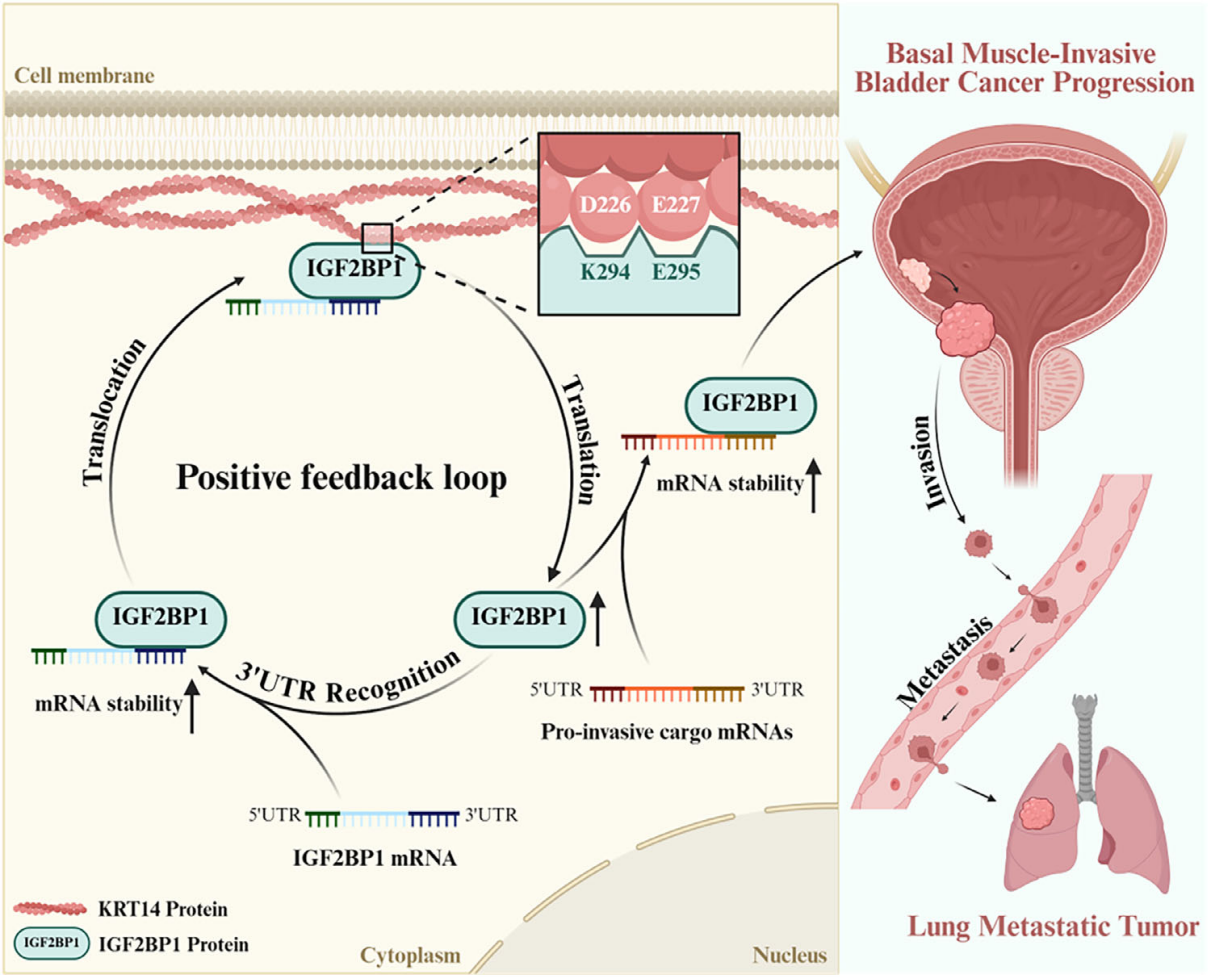
Mechanistic model illustrating the role of the KRT14‐IGF2BP1 axis in BMIBC invasive progression.

To assess the biological relevance of the KRT14‐IGF2BP1 interaction interface, we mutated KRT14 residues D226 and E227. Compared with KRT14‐WT cells, cells expressing KRT14‐MUT exhibited significantly reduced lung metastatic colonization in nude mice (Figure 6L–N) and diminished invasive capacity in *vitro* (Figure S20A–G), indicating that the D226/E227‐dependent interaction with IGF2BP1 is required for KRT14‐driven invasion and metastatic colonization. Together with the structural modeling data, these findings identify the LDEL segment of KRT14 (225–228 aa) and the GKEG loop of IGF2BP1 (293–296 aa) as a functionally important interaction interface in BMIBC progression. A schematic model summarizing this mechanism is presented in Figure 7.

## 3 Discussion

Early studies established that basal and luminal cells are defined by distinct keratin expression patterns, with basal cells predominantly expressing KRT5 and KRT14 [63], and luminal cells expressing KRT8 and KRT18 [64, 65]. These keratin profiles have long been used to determine tumor origin and molecular subtypes. Among them, KRT14 has emerged as both a diagnostic marker and a potential indicator of tumor progression [66, 67, 68]. Previous studies have linked *Krt14^+^* basal‐like cells to distant metastasis in breast cancer and to invasive potential in ovarian and lung carcinomas [69, 70, 71, 72, 73, 74]. Consistently, in BCa, KRT14 functions as a downstream effector of TRIM29, contributing to tumor invasion [75, 76]. Moreover, impaired KRT14 degradation resulting from TGM3 loss has been shown to promote poorly differentiated cutaneous squamous cell carcinoma [77]. Collectively, these findings underscore the multifaceted roles of KRT14 in tumor biology and highlight its potential as both a biomarker and therapeutic target across multiple cancer types.

In the present study, we investigated the biological and clinical significance of KRT14 in BCa progression. ScRNA‐seq analysis of nine human BCa samples revealed that the KRT14‐high cell cluster exhibited both EMT and CSC characteristics, suggesting a pivotal role of KRT14 in driving malignant phenotypes. Both human and BBN‐induced mouse bladder tumors displayed markedly elevated KRT14 expression compared with their respective normal counterparts. Consistent results across TCGA‐BLCA, GEO, and UC‐GENOME demonstrated that KRT14 was significantly upregulated in BMIBC and that higher KRT14 expression correlated with poorer overall survival. In our clinical cohort, IHC analysis further confirmed stronger KRT14 staining in MIBC than in NMIBC, and patients with high KRT14 levels exhibited unfavorable prognostic outcomes. In the BBN‐induced BMIBC mouse model, Krt14 expression progressively increased during tumor development, while urothelial‐specific knockout of Krt14 markedly suppressed tumorigenesis and invasion. Together, these findings establish KRT14 not only as a subtype‐defining marker but also as a functional driver of BMIBC progression.

By integrating in vivo conditional knockout modeling, histological assessment, and scRNA‐seq analyses, we identified the KRT14‐IGF2BP1 axis as a central driver of malignant progression in BMIBC. Importantly, ablation of Krt14 via *Upk2*‐Cre did not perturb urothelial homeostasis under physiological conditions, as evidenced by the absence of histopathological or barrier defects. These findings indicate that KRT14 is largely dispensable for steady–state tissue maintenance, minimizing potential confounding effects of developmental abnormalities on tumor phenotypes. Previous studies have shown that *Krt14* ^+^ cells define a basal progenitor compartment in the bladder that contributes to both stress‐responsive epithelial repair and tumor initiation [15]. Building on this framework, we hypothesized that under chronic BBN‐induced carcinogenic stress, KRT14 supports the activation and expansion of these progenitors, thereby providing a favorable cellular context for malignant evolution. To test this hypothesis, we experimentally limited Krt14 availability and observed that tumor onset was delayed and malignant progression constrained. Because *Upk2*‐Cre predominantly targets differentiated urothelial layers rather than basal progenitors, Krt14 deletion produced mosaic “Krt14‐low” tumors rather than complete ablation. Although a fraction of Krt14‐CKO mice eventually developed invasive lesions after prolonged BBN exposure, these tumors likely originated from residual *Krt14* ^+^ basal cells that escaped recombination, highlighting the essential role of this lineage in facilitating malignant transformation. Mechanistically, scRNA‐seq and pseudotime trajectory analyses revealed that in WT tumors, *Igf2bp1^+^ Krt14^+^* basal cells give rise to EMT‐like progeny, maintaining a continuum of epithelial states associated with aggressive disease. In Krt14‐deficient tumors, this differentiation axis collapses, accompanied by the loss of *Igf2bp1^+^* basal population and their downstream EMT‐like cells. Overall, these findings identify the KRT14‐IGF2BP1 axis as a key molecular circuit that preserves epithelial plasticity and enables malignant progression in BMIBC.

In most tumor types, IGF2BP1 is markedly upregulated, and its expression correlates with poor patient prognosis [37]. Functionally, IGF2BP1 promotes tumor progression through multiple oncogenic processes, including enhanced proliferation [78] and immune evasion [79], and, most prominently, metastasis [80]. Mechanistically, IGF2BP1 stabilizes mRNAs encoding pro‐metastatic regulators such as *SNAI2* [81], *CD44* [82], *ACTB* [83], and *CTNNB1* [84], thereby facilitating EMT, cytoskeletal remodeling, and increased tumor cell motility. These multifaceted activities highlight IGF2BP1 as a promising therapeutic target, as its inhibition could disrupt several key pathways underlying tumor aggressiveness. Notably, its expression is largely restricted to embryonic and malignant tissues and is rarely detected in normal adult cells [85], supporting the notion that IGF2BP1 upregulation represents a tumor‐associated reactivation event during oncogenesis. Although its downstream oncogenic functions have been extensively characterized, the upstream regulatory mechanisms governing IGF2BP1 expression in BCa remain poorly understood. Given these observations, we hypothesized that KRT14 may act as an upstream regulator of IGF2BP1 in BCa.

Our data support a model in which KRT14 directly interacts with IGF2BP1 to establish a self‐reinforcing regulatory circuit that sustains IGF2BP1‐dependent pro‐invasive gene programs in BMIBC. IGF2BP1 contains RRMs and KH domains, with the KH2 domain functioning as a dual interface for RNA binding and protein interaction. Co‐IP and mutagenesis analyses identified a specific contact between KRT14 residues D226/E227 and IGF2BP1 residues K294/E295 within the KH2 domain, and this structural coupling enhanced IGF2BP1's affinity for its own mRNA, thereby amplifying IGF2BP1 protein abundance and sustaining oncogenic signaling. Mutation of KRT14 D226/E227 disrupted this interaction and abolished IGF2BP1 autoregulation, confirming that this feedback circuit depends on the integrity of the KRT14‐IGF2BP1 interface. Beyond post‐transcriptional feedback, the spatial dimension of this interaction appears critical. Specifically, we observed that in KRT14‐overexpressing cells, *IGF2BP1* mRNA is prominently enriched at the cytoplasmic periphery and protrusive regions. Given the cytoskeletal localization of KRT14, its direct interaction with IGF2BP1, and the capacity of IGF2BP1 to bind and stabilize its own mRNA, we propose that the KRT14‐IGF2BP1 complex may guide the polarized accumulation of *IGF2BP1* mRNA. This spatial organization may facilitate local activation of invasion‐associated RNA programs, consistent with previous studies showing that the precise accumulation of driver RNAs at protrusive regions supports invasive phenotypes [61, 86]. This may also explain why KRT14 loss had a stronger effect on invasion than on migration. Compared with migration, invasion requires tumor cells to engage with ECM barriers and activate matrix‐interacting programs. In line with this distinction, KRT14 enhanced IGF2BP1‐dependent regulation of pro‐invasive transcripts such as *VCAN* and *ZEB1* and promoted redistribution of *VCAN* mRNA toward peripheral cytoplasmic regions. Thus, the KRT14‐IGF2BP1 axis appears to preferentially support ECM‐related and EMT‐associated programs involved in invasion.

Despite these advances, several limitations remain. First, KRT5 may also contribute to the phenotypes observed here. Although KRT5 and KRT14 can form heterodimers in stratified epithelia, lineage‐tracing and scRNA‐seq studies suggest that their expression is not always concordant during urothelial development [15, 87, 88]. Thus, the contribution of KRT5 to the effects observed after Krt14 loss remains unclear. Further analysis of KRT5 expression and KRT5/KRT14‐defined basal‐cell states in Krt14‐deficient tumors will be needed to address this issue. Second, although *Krt14* ^+^ basal cells are known to contribute to epithelial renewal and injury‐induced regeneration [15], whether complete Krt14 loss alters these processes remains unresolved. Future studies using basal cell‐specific deletion models combined with lineage tracing and injury‐repair assays will be needed to determine how KRT14 affects urothelial repair and repair‐associated tumorigenesis.

Taken together, our study identifies the KRT14‐IGF2BP1 axis as a mechanistic link between basal plasticity and aggressive BMIBC progression. Through direct structural interaction, post‐transcriptional amplification, and spatial mRNA localization, this pathway sustains pro‐invasive gene programs and may represent a potential therapeutic vulnerability.

## 4 Materials and Methods

### 4.1 Ethics Approval

All experiments involving clinical specimens were approved by the Ethics Committee of the First Affiliated Hospital of Wenzhou Medical University. The tumor tissue samples used in this study were obtained from BCa patients treated at the First Affiliated Hospital of Wenzhou Medical University (Zhejiang, China). A total of 98 histologically and pathologically confirmed specimens were collected (Table S3). All animal experiments were conducted in accordance with the regulations of the Animal Experimental Center of Wenzhou Research Institute, University of Chinese Academy of Sciences.

## Author Contributions

C.H. and S.H. performed conceptualization. S.H. and Z.Z. performed data curation. S.H., Z.Z., and Z.W. performed formal analysis. C.H. and S.H. performed funding acquisition. S.H., L.He, Y.L., L.Huang, L.Y., L.H., and X.L. performed investigation. C.H., S.H., Q.X., and X.H. performed methodology. C.H., S.H., Y.Z., and J.L. performed project administration. C.H., W.C., X.S., G.W., J.C., Q.X., and B.L. acquired resources. Z.Z., Z.W., and Y.W. acquired software. C.H., W.C., X.S., G.W., and J.C. performed supervision. S.H., Z.Z., Z.W., and Y.W. performed validation. S.H., Z.Z., and Q.X. performed visualization. S.H. and Z.Z. wrote original draft. C.H., S.H., and Q.X. wrote review and editing.

## Conflicts of Interest

The authors declare no conflicts of interest.

## Supporting information




**Supporting File 1**: advs75900‐sup‐0001‐SuppMat.docx.

**Supporting File 2**: advs75900‐sup‐0002‐TableS1‐S3.

**Supporting File 3**: advs75900‐sup‐0003‐VideoS1.mp4.

## Data Availability

All data needed to evaluate the conclusions in the paper are present in the paper and/or the Supporting Materials.
