# Supplementary material for: KRT14 Drives Basal Muscle‐Invasive Bladder Cancer Progression and Lung Metastasis by Directly Binding to and Stabilizing IGF2BP1
Source: Adv Sci (Weinh). 2026 Jun 9;13(49):e75900. doi: 10.1002/advs.75900 (PMC13336978; doi:10.1002/advs.75900)
Supplement: Supplementary file 1 [file ADVS-13-e75900-s003.docx]

Supporting Information for

**KRT14 Drives Basal Muscle-Invasive Bladder Cancer Progression and Lung Metastasis *by* Directly Binding to and Stabilizing IGF2BP1**

Shirui Huang, Zhihan Zhou, Qipeng Xie, Zheng Wang, Lijiong He, Yutong Liu, Lijuan Huang, Liuxian Ye, Limeng Hu, Baokun Li, Yang Wang, Xiaohui Hua, Xuelei Liu, Yunping Zhao, Jingxia Li, Jinfei Chen^*^, Guiying Wang^*^, Xian Shen^*^, Wei Chen^*^ and Chuanshu Huang^*^

Corresponding authors

Jinfei Chen: jinfeichen650909@wmu.edu.cn

Guiying Wang: wangguiying@hebmu.edu.cn

Xian Shen: shenxian@wmu.edu.cn

Wei Chen: chenweiuro@wmu.edu.cn

Chuanshu Huang: huangchuanshu@ojlab.AC.cn

**The file includes:**

Supporting information text

Figures S1 to S20

Legends for tables S1 to S3

Legend for video S1

References

**Other supporting materials for this manuscript include the following:**

Tables S1 to S3

Video S1

**Supporting** **Information Text**

**Materials and Methods**

**Plasmids and antibodies**

The sgRNA plasmids targeting KRT14 (sgRNA-1: 5'- TCATCCTCCCGCTTCTCCTC -3'; sgRNA-2: 5'- AGCGGGAGGATGAGACAGAC -3'; sgRNA-3: 5'- CAGCTTTGGTAGTGGCTTT -3') and its lentiCRISPR v2 vector were purchased from Public Protein/Plasmid Library (Nanjing, China). The plasmids pET263-pUC57 24×MS2V7 (Plasmid #140705), phage UbiC NLS HA stdMCP stdHalo (Plasmid # 104999), FU-tet-o-hOct4 (Plasmid #19778), FUdeltaGW-rtTA (Plasmid #19780), pMD2.G (Plasmid #12259) and psPAX2 (Plasmid #12260) were all sourced from Addgene. HA-KRT14, HA-KRT14-N-term, HA-KRT14-C-term, HA-KRT14-D226A, HA-KRT14-E227A, HA-KRT14-D226A/E227A plasmids were cloned into the pLVX-HA vector, IGF2BP1-3×Flag, IGF2BP1-3×Flag-RRM1-2, IGF2BP1-3×Flag-KH1-4, IGF2BP1-3×Flag-KH1-2, IGF2BP1-3×Flag-KH3-4, IGF2BP1-3×Flag-KH2AA, IGF2BP1-3×Flag-KH1DD/KH2DD and IGF2BP1-3×Flag-KH3EE/KH4EE plasmids were constructed in the pEnCMV-3×Flag vector; IGF2BP1-promoter plasmid was cloned into the pGL3-Basic luciferase reporter vector; IGF2BP1-3'UTR-Short, IGF2BP1-3'UTR-Medium, IGF2BP1-3'UTR-Long, IGF2BP1-3'UTR-Long1 and IGF2BP1-3'UTR-Long2 plasmids were cloned into the pMIR-Reporter luciferase reporter vector. The sgRNA targeting IGF2BP1 (sgRNA: 5'- AATGGCACCCAC ATACTGGG -3') was constructed in the pX459M vector. Antibodies specific against HA (3724S), Flag (14793S) and ZEB1 (3396S) were obtained from Cell Signaling Technology (Beverly, MA, USA). An antibody specific against Versican (S0B6760) was from STARTER (Hangzhou, China). An antibody specific against GFP (Sc-9996) was purchased from Santa Cruz Biotechnology (Santa Cruz, CA, USA). Antibodies specific against IGF2BP1 (22803-1-AP) and GAPDH (10494-1-AP) were obtained from Proteintech (Wuhan, China). An antibody specific against KRT14 (ab7800) was from Abcam (Cambridge, UK).

**Cell culture and transfection**

SV-HUC-1 and U5637 cells were cultured in 1640 medium (SIGMA, 11875-093), J82 cells were maintained in MEM medium (Gibco, 11095-080), T24T cells were cultured in DMEM: F12 medium (Gibco, 10565‐018), and UMUC3 and HEK293 cells were maintained in DMEM medium (Gibco, 11995‐065). All media contained 10% FBS, except for the DMEM: F12 medium which contained 5% FBS (Gibco; 10437-028). All cells were cultured in an incubator at 37°C with 5% CO_2_.

The stable ectopic expression of KRT14 and the knockout of KRT14 were respectively achieved through lentiviral transduction. The pMD2.G, psPAX2 and target plasmids were added to HEK293 cells in a specific ratio, with PolyJet™ DNA In Vitro Transfection Reagent (SignaGen Laboratories, SL100688) used for transfection. After 48 hours of transfection, viral supernatants were collected and added to BCa cells. Puromycin selection was employed on transfected cells to obtain stable cell lines overexpressing KRT14 as well as cells with KRT14 knockout.

Other cell models were constructed using liposome-mediated transfection. The target plasmids were introduced into the cells using a transfection reagent, and stable transfectants were selected with appropriate antibiotics.

**The construction of the BBN-induced BMIBC mouse model**

Experimental group mice were fed drinking water containing 0.05% BBN, while control group mice were given additive-free drinking water, with changes every three days. After 15 weeks of treatment, hematuria occurrence in the mice was closely monitored. By the 23rd week, approximately 75-100% of the mice in the BBN-treated group had developed MIBC. The bladders of mice from each group were photographed and documented, and some tissues were embedded for subsequent HE staining and IHC analysis.

**Genetically engineered mouse model**

The Krt14-CKO mice were generated by crossing *Krt14*^lox/lox^ mice (GemPharmatech Co., Ltd, T009976) with *Upk2*-Cre mice (GemPharmatech Co., Ltd, T050812), and the targeted deletion of the *Krt14* allele and the expression of the *Cre recombinase* allele were confirmed by PCR genotyping. The primers utilized in this study were as follows: *Krt14* (forward: 5′-CTTGTTCCTCCAATGGGTGTTTG-3′ and reverse: 5′-GCTGAGAAATAGGCTGTCTCCTCTG-3′); *Upk2*-Cre (forward1: 5′-GTCCTGTTCAATCATCTCTGCACTAG-3′ and reverse1: 5′-GGAGCATCTTCCAGGTGTGTTCA-3′); (forward2: 5′-CTAAGCTGTGAACCACAGGAGCTAC-3′ and reverse2: 5′- CGTGGGTCAGCCTTTATTGTGAG-3′). The histopathology of Krt14-CKO and WT mice was evaluated by professional pathologists who were blinded to the genotypes.

**Cell migration and invasion assay**

Migration chambers (Corning, 353097) and Matrigel matrix (Corning, 354234) were used to assess cell invasion and migration capabilities. Chambers coated with Matrigel matrix were employed to evaluate cell invasion, while those without Matrigel matrix assessed cell migration. An appropriate number of cells were seeded into the chambers and cultured for 24 hours. After culturing, the old medium was removed, and the chambers were washed with PBS. Subsequently, cells were fixed with 4% paraformaldehyde (PFA), permeabilized with methanol, and stained with Giemsa. Internal cells were removed with a cotton swab, and images were captured for cell counting.

**Lung metastatic assay**

All animal experiments were approved by the Ethics Committee of the Wenzhou Research Institute, University of Chinese Academy of Sciences. Female BALB/c nude mice, aged 3-4 weeks, were purchased from Vital River (Beijing, China) and housed in the SPF-grade animal facility at the Animal Experimental Center of the Wenzhou Research Institute, University of Chinese Academy of Sciences. After one week of adaptation, the mice were randomly assigned to groups and ear-tagged for identification. Experimental group cells and control group cells were injected into the mice via the tail vein at a concentration of 3×10^6^ cells/100 μl PBS. After 8 weeks, the mice were euthanized, and lung tissues were harvested. The metastatic foci were photographed after fixation with picric acid and counted. HE staining was performed for the pathological analysis of the metastatic foci.

**Immunohistochemical staining experiment (IHC)**

Immunohistochemical staining was performed on paraffin-embedded mouse bladder tissue sections using a commercial staining kit (Vazyme, HC301-01) according to the manufacturer's instructions. Briefly, the sections were sequentially subjected to antigen retrieval, incubation with Hydrogen Peroxide Blocking Reagent to quench endogenous peroxidase activity, primary antibody incubation, and incubation with HRP Polymer. DAB was applied to visualize target protein expression, and hematoxylin was used for nuclear counterstaining. Microscopic images were acquired, and the staining intensity was quantified by calculating the integrated optical density normalized to area (IOD/area).

**Cell Immunofluorescence Assay**

Cells were seeded onto coverslips placed in 24-well plates and cultured until reaching appropriate density. The old medium was discarded, and the cells on the coverslips were washed 2-3 times with PBS. The cells were then sequentially fixed with 4% PFA, permeabilized with Triton X-100 (Beyotime, P0096), and blocked with quick-blocking solution (Beyotime, P0260). Incubation was performed with IP-grade primary antibodies, followed by fluorescent secondary antibodies, including Goat Anti-Rabbit IgG H&L (Abcam, ab150077) and Goat Anti-Mouse IgG H&L (Abcam, ab150115). DAPI staining was then conducted. Finally, the coverslips were mounted onto glass slides, and images were acquired using a laser scanning confocal microscope (LSM980, ZEISS).

**Western blot analysis**

Cells reaching appropriate density and exhibiting good cellular status were used for protein sample preparation. Cell lysis was performed on ice using cell lysis buffer, and lysates were promptly collected. Following sonication, protein concentration was determined using the NanoDrop One (Thermo Scientific), and all samples were adjusted to the same concentration. After complete protein separation by SDS-PAGE, the proteins were transferred onto PVDF membranes. The membranes were blocked with 5% non-fat milk, followed by sequential incubation with primary and secondary antibodies. Imaging was conducted using the fluorescence imaging system of Typhoon FLA 7000 (GE Healthcare).

**RNA stability assay**

Appropriate cell numbers were seeded into six-well plates and allowed to adhere and spread. After reaching confluence, the cells were serum-starved in medium containing 0.1% FBS for 12 hours. Subsequently, the cells were treated with complete medium containing 10 µg/ml Act D (psaitong, A6001) at different time points. At the final time point, all cells were collected uniformly. Total RNA was then manually extracted, followed by cDNA synthesis through reverse transcription, and further analysis using qPCR.

**Immunoprecipitation (IP)**

The plasmids containing the deleted domain constructs, control plasmids, and full-length plasmids were separately transfected into U5637 cells. After 8 hours of transfection, the medium was replaced, and the cells were further cultured for 24-36 hours. The old medium was discarded, and the cells were washed twice with pre-cooled PBS. IP lysis buffer (Cell Signaling Technology, 9803S) was added, and the cells were lysed on ice for 10 minutes before collection. Subsequently, the lysates were centrifuged at 4°C and 14000g for 10 minutes, and clear supernatant was taken as input samples. The protein concentration in the lysates was determined using the BCA method. After ensuring equal protein amounts in each sample, 20 μl of anti-HA-tag mAb-Magnetic Beads (MBL, M180-11) or the corresponding Mouse IgG2b (isotype control)-Magnetic Beads (MBL, M077-11), and anti-DDDDK-tag mAb-Magnetic Beads (MBL, M185-11R) or the corresponding Mouse IgG2a (isotype control)-Magnetic Beads (MBL, M076-11), were added to each sample and rotated overnight at 4°C. The magnetic beads were separated using a magnetic rack and washed multiple times with IP lysis buffer. Cell lysis buffer was added to the magnetic beads, and proteins were completely eluted by heating to 100°C. Protein immunoblotting experiments were performed using HA antibody or Flag antibody.

**RNA-Protein Immunoprecipitation (RIP)**

As previously described [1], a specified number of cells were prepared and lysed using polysome lysis buffer. The cell lysates were centrifuged at 14,000g for 10 minutes at 4°C, and the supernatants were transferred and evenly distributed into new EP tubes. These were incubated overnight with IgG, primary antibody, and agarose beads A/G (Santa Cruz, sc-2003) sequentially. Subsequently, the agarose beads were washed with NET2 buffer to remove nonspecific bindings. Finally, RNA was extracted using Trizol, followed by reverse transcription and qPCR analysis.

**Live-cell RNA Visualization Using the MS2-MCP System**

The endogenous 5'UTR and IGF2BP1 coding region were cloned upstream of 24×MBSV7 stem-loop repeats (from Addgene #140705), positioned between the CDS and the full-length IGF2BP1 3'UTR. This configuration maintains native UTR-mediated post-transcriptional regulation while allowing MS2 labeling of IGF2BP1 transcripts. The resulting reporter cassette replaced the original hOct4 insert in the FU-tet-o-hOct4 plasmid, retaining its doxycycline-responsive regulatory elements and enabling doxycycline-inducible expression together with FUdeltaGW-rtTA. The final construct is referred to as the Tet-inducible IGF2BP1-24×MBSV7 reporter.

The Tet-inducible IGF2BP1-24×MBSV7 reporter, FUdeltaGW-rtTA, and the MCP-Halo plasmid (Addgene #104999) were delivered into KRT14-overexpressing U5637 cells and their corresponding control cells via lentiviral transduction. Detailed procedures for lentiviral production and infection are described in Cell Culture and Transfection under the Materials and Methods section. After confirming successful plasmid expression, cells were treated with doxycycline (2 µg/mL) for 24 h to induce reporter expression. To label MCP-Halo, cells were incubated with 200 nM JFX646 HaloTag ligand (Promega, GA1120) for 2 h prior to live-cell imaging.

Live-cell single-mRNA imaging was performed using a SpinSR spinning-disk confocal microscope equipped with a 60×/1.50 NA TIRF oil-immersion objective and a stage-top incubator maintained at 37 °C with 5% CO₂. MCP-Halo was excited using a 640-nm laser. Time-lapse images were acquired every 20 s for ~20 min using imaging settings optimized for single-molecule detection.

**RNA Fluorescence in Situ Hybridization (RNA-FISH)**

RNA-FISH was performed using a Fluorescent In Situ Hybridization Kit (RiboBio, C10910) according to the manufacturer’s protocol. Briefly, cells were seeded on glass coverslips in 24-well plates and cultured to 60%-70% confluence. The cells were fixed with 4% PFA at room temperature, permeabilized with precooled permeabilization buffer, and hybridized with specific RNA-FISH probes against VCAN or 18S overnight at 37℃ in the dark. After hybridization, the cells were washed sequentially with hybridization wash buffers at 42°C and then with PBS. Nuclei were counterstained with DAPI, and fluorescence images were acquired using a confocal microscope (Leica, STELLARIS 5).

**Bulk RNA-seq**

Total RNA was extracted from U5637 BCa cells overexpressing HA-KRT14 and their vector control. RNA integrity was assessed using the Agilent Bioanalyzer 2100 system, followed by library preparation and sequenced at Illumina NovaSeq platform. Clean reads were aligned to the human reference genome (GRCh38) using HISAT2, and differential expression analysis was performed using DESeq2 with |log_2_FoldChange| ≥ 1 and padj < 0.05 set as significance thresholds. Data analysis was provided by Novogene Co., Ltd (Tianjin, China).

**RT-PCR and qPCR**

Total RNA was manually extracted from cells stored in TRIzol reagent (Invitrogen, 15596018), and cDNA was synthesized following the instructions of the reverse transcription kit (Takara, RR037A). The qPCR steps were described in detail in our previous studies [2]. The primers utilized in this study were as follows: human *IGF2BP1* (forward: 5′- CAGGAGATGGTGCAGGTGTTTATCC-3′ and reverse: 5′-GTTTGCCATAGATTCTTCCCTGAGC-3′); human *GAPDH* (forward: 5′-GGAGCG AGATCCCTCCAAAAT-3′ and reverse: 5′-GGCTGTTGTCATACTTCTCATGG-3′); human endogenous *IGF2BP1-*Total (forward: 5′-ACGGGCAGAAATCGAGAGTG-3′ and reverse: 5′-GGCTGAGAGATCAGGGTTCC-3′); human endogenous *IGF2BP1-*Medium (forward: 5′-TGCTTACAGTATTGACTCAAGGGAA-3′ and reverse: 5′-TCCCCATTTTCCCCTCTTCTT-3′); human endogenous *IGF2BP1-*Long (forward: 5′-CAGCACAGCCTGTCACAGTA-3′ and reverse: 5′-GTCCCGTACCCCGATAGA GA-3′); human *VCAN* (forward: 5′-AACGGCTTTGACCAGTGCGA-3′ and reverse: 5′-ATCAGGGGGAGGGAAGCCTG-3′); human *ZEB1* (forward: 5′-TTACACCTTTGCATACAGAACCC-3′ and reverse: 5′-TTTACGATTACACCCAGACTGC-3′); mouse *Krt14* (forward: 5′-AGCGGCAAGAGTGAGATTTCT-3′ and reverse: 5′-CCTCCAGGTTATTCTCCAGGG-3′); and mouse *Gapdh* (forward: 5′-GGAGAG TGTTTCCTCGTCCC-3′ and reverse: 5′-ATGAAGGGGTCGTTGATGGC-3′).

**Tissue Preparation and scRNA-seq**

This study included four mouse sample types for scRNA-seq analysis: WT-Vehicle, WT-BBN, Krt14-CKO-Vehicle, and Krt14-CKO-BBN. Bladder tissues from these groups were processed according to the single-cell preparation protocol recommended by Novogene (Shanghai, China). Tissues were gently minced into ~1 mm³ pieces and enzymatically dissociated to obtain single-cell suspensions. The dissociated cells were loaded into Chromium microfluidic chips and barcoded using the Chromium Controller (10x Genomics) with Single Cell 3′ v3 chemistry. cDNA synthesis and library construction were performed using the Chromium Single Cell 3′ reagent kit (10x Genomics), and sequencing was conducted on an Illumina NovaSeq platform.

**Bioinformatics Analysis**

ScRNA-seq datasets (GSE267718 and GSE222315) were obtained from the GEO database. After loading the expression matrices, quality control and downstream analyses were conducted using the Seurat package (version 5.1.0) in R (version 4.4.1). Cells with fewer than 200 detected genes, more than 6000 detected genes, or mitochondrial gene percentages exceeding 10% were excluded. Normalization was performed using the LogNormalize method, and highly variable genes were identified using the FindVariableFeatures function. The data were subsequently scaled with the ScaleData function while regressing out mitochondrial gene content to reduce technical bias. Doublets were identified and removed using the DoubletFinder and cell clusters were generated using the FindClusters function in Seurat, followed by visualization with t-SNE or UMAP. Cluster annotation was based on canonical marker gene expression profiles. After quality control and doublet removal, 31,096 high-quality cells were retained for downstream analyses.

For the GSE267718 dataset, eight major cell populations were annotated based on canonical marker gene expression, including B cells (*CD79A, CD19*), endothelial cells (*PECAM1*), fibroblasts (*COL1A1*), megakaryocytes (*PF4, PPBP*), monocytes/macrophages (*LYZ, CD14, MRC1, CD68*), plasma cells (*CD79A, IGHG1*), T cells (*CD3D, CD3E*), and urothelial cells (*EPCAM, UPK2, UPK1A*). Using the same analytical pipeline, we processed the GSE222315 dataset and identified 690 normal urothelial cells. Differential gene expression analysis was subsequently performed to compare tumor-derived and normal urothelial cells.

The same scRNA-seq data processing and analysis workflow was applied to mouse bladder samples. Based on canonical marker gene expression, we annotated ten cell types, including B cells (*Cd79a*), endothelial cells (*Cldn5, Pecam1*), epithelial cells (*Upk1b, Upk3a, Epcam*), fibroblasts (*Col6a1, Col6a2*), macrophages (*Cd68, Cd86*), mdsc (*S100a8, S100a9*), natural killer cells (*Nkg7*), schwann cells (*Sox10, Fabp7*), smooth muscle cells (*Acta2*), and T cells (*Cd3d, Cd3g*). Differential gene expression analysis was subsequently performed to compare urothelial cells from BBN-induced tumors with those from vehicle-treated controls.

As there is not yet a validated EMT signature specific to BCa, we prospectively generated a gene list based on established EMT markers curated from EMTome [3]. The EMT scoring approach was adapted from a previously reported signature-based scoring methodology [4], while the epithelial and mesenchymal gene sets were specifically selected for BCa in this study. The EMT score was calculated by adding the sum of the log2 Z scores of six mesenchymal genes (MMP9, SNAI2, VIM, TWIST2, FN1, INHBA) and subtracting the sum of the log2 Z scores of five epithelial genes (CDH1, CLDN4, EPCAM, MAL2, and ST14). In this scoring system, higher scores indicate a more mesenchymal-like state, whereas lower scores reflect a more epithelial-like phenotype. Stemness scores were assessed using CytoTRACE2.

Unsupervised trajectory analysis was performed using Monocle3 (v1.3.7) to infer differentiation pathways among tumor cell subtypes through pseudotime modeling. CytoTRACE2 (v1.1.0) was integrated to predict differentiation order and stemness characteristics, thus facilitating the determination of an appropriate trajectory root and reducing uncertainty in lineage initiation.

Sequencing data from the TCGA-BLCA cohort were utilized to analyze KRT14 expression and its association with clinical outcomes (https://tcga-data.nci.nih.gov/). According to the classification described in the article by A Gordon Robertson [5], the subtypes of BCa were distinguished. In addition, KRT14 expression in BCa was further evaluated using GEO datasets (https://www.ncbi.nlm.nih.gov/geo/), and prognostic relevance was validated in the UC-GENOME cohort via cBioPortal (<https://www.cbioportal.org/study/summary?id=blca_bcan_hcrn_2022>).

**Evaluation of ICI responsiveness-related signatures**

IFN-γ and T cell-inflamed gene expression profile (GEP) signatures were analyzed using scRNA-seq data to evaluate immune activation and potential ICI responsiveness. Human gene sets reported by Ayers et al. [6]were converted to mouse orthologs for analysis in the BBN-induced MIBC model. The mouse IFN-γ signature included Ifng, Stat1, Cxcl9, Cxcl10, and Ido1, and the mouse T cell-inflamed GEP included Ccl5, Cd27, Cd274, Cd8a, Cxcl9, Cxcr6, Ido1, Lag3, Nkg7, Pdcd1lg2, Psmb10, Stat1, and Tigit. Signature scores were calculated for each cell using the AddModuleScore function in Seurat. Differences between WT and Krt14-CKO groups were analyzed using the Wilcoxon rank-sum test. Violin plots were generated using ggplot2 and ggpubr.

**Protein-protein interaction prediction**

Protein complex interactions between KRT14 and IGF2BP1 were predicted using DMFold-Multimer (https://zhanggroup.org/DMFold/). The top-ranked model was visualized in PyMOL, and putative interfacial hydrogen bonds were identified using default hydrogen-bond settings.

**Statistical analysis**

The expression levels of KRT14 in basal and luminal subtypes of MIBC were analyzed, along with the associated prognostic survival analysis. Prognostic survival analysis was performed using the Kaplan-Meier method for plotting and the log-rank test for statistical analysis.

GraphPad Prism 6.0 (San Diego, USA) was used for all data analysis and graph generation. Data are expressed as mean ± SD. Statistical comparisons were made using the 2-tailed unpaired Student’s t test. Correlation was assessed using Pearson's correlation test. P < 0.05 indicated significant differences.

**Figures**

**Fig.S1. scRNA-seq analysis of human BCa.** (A) t-SNE plot showing the distribution of distinct cell types in nine human BCa tissues (GSE267718) based on scRNA-seq data, with each cell type color-coded. (B) t-SNE visualization displaying the expression patterns of representative marker genes across all cell types. (C) t-SNE plot showing reclustering of epithelial cells extracted from panel (A), with cells color-coded according to their origin from NMIBC or MIBC samples. (D) Dynamic gene expression module clustering of epithelial cell clusters, revealing temporal patterns and enriched biological processes. (E) t-SNE plot showing the distribution of distinct cell types identified in four human normal bladder tissues (GSE222315) based on scRNA-seq data, with each cell type color-coded. (F) t-SNE visualization displaying the expression patterns of representative signature genes in epithelial cells extracted from panel (E). (G) t-SNE plot showing reclustering of epithelial cells extracted from panels (A) and (E), with cells color-coded according to their origin from normal or tumor samples.

**Fig.S2.** **Expression dynamics of canonical basal markers during bladder carcinogenesis in BBN-treated mice.** (A) Schematic illustration of the BBN-induced spontaneous BMIBC model in mice. Mice were administered 0.05% BBN in drinking water for 25 weeks, with fresh BBN solution replaced twice weekly. (B to D) Representative IHC and HE staining images, along with quantitative analysis of Krt5 and Cd44 protein expression in the urothelium of WT mice at different time points following BBN exposure. Data are expressed as the mean ± SD. The symbol (*) indicates a statistically significant increase in Krt5 and Cd44 expression in the BBN-treated group compared with the vehicle group, or between consecutive time points during BBN induction (p < 0.05).

**Fig.S3. scRNA-seq analysis of the BBN-induced mouse BCa model.** (A) t-SNE plot showing dimensionality reduction and clustering of cells from scRNA-seq data of bladder tissues from vehicle and BBN-treated mice. (B) Heatmap displaying the expression of representative marker genes across all identified cell clusters. (C) t-SNE plot showing the distribution of distinct cell clusters identified from scRNA-seq data of bladder tissues from two biological replicates of vehicle-treated and BBN-treated mice, with each cluster color-coded. (D) Proportional analysis of distinct cell clusters identified from scRNA-seq data of bladder tissues from two biological replicates of vehicle-treated and BBN-treated mice. (E) Heatmap showing the expression patterns of key marker genes in distinct cell types. (F) Bubble plot showing representative DEGs in bladder epithelial cells between vehicle and BBN-treated mice.

**Fig.S4.** **Krt14 expression dynamics during bladder carcinogenesis in BBN-treated mice.** (A) Quantitative PCR analysis of *Krt14* expression in the bladder epithelium from vehicle- and BBN-treated mice. Each group included one pooled bladder epithelial sample generated from five individual mice, with three technical replicates shown. (B to C) Representative IHC and HE staining images, along with quantitative analysis of Krt14 protein expression in the urothelium of WT mice at different time points following BBN exposure. Data are expressed as the mean ± SD. The symbol (*) indicates a statistically significant increase in Krt14 expression in the BBN-treated group compared with the vehicle group, or between consecutive time points during BBN induction (p < 0.05).

**Fig.S5. Validation of KRT14 expression in BCa across multiple databases.** (A) Comparison of KRT14 mRNA expression between BCa tissues and paired adjacent normal tissues in the TCGA dataset (n = 19). (B) Analysis of KRT14 mRNA expression in unpaired BCa and normal tissues from TCGA cohort. (C) Evaluation of KRT14 expression in BCa using the GSE13507 dataset from GEO. (D) Association between KRT14 expression and tumor grade in the TCGA cohort. (E) Distribution of classical molecular subtypes between KRT14-high and KRT14-low groups in the TCGA-BLCA dataset. (F) Comparative analysis of KRT14 expression across molecular subtypes of MIBC using TCGA data. (G) Differential KRT14 expression among MIBC subtypes in the GSE87304 microarray dataset. (H) Kaplan-Meier analysis of overall survival according to KRT14 expression levels in the TCGA cohort. (I) Survival analysis among different MIBC subtypes within TCGA dataset. (J) Kaplan-Meier analysis of overall survival in BCa cases selected from the UC-GENOME cohort, stratified by KRT14 expression levels. (K) Representative IHC staining of KRT20 in human BCa tissues with high versus low KRT14 expression. Data are expressed as the mean ± SD.

**Fig.S6. Characterization of the conditional knockout Krt14 mouse model.** (A) Genotyping results confirming conditional knockout of Krt14 in mice. (B to C) Transcriptional and protein expression levels of Krt14 in the urothelium of untreated Krt14-CKO mice. (D) Representative histological images showing urothelial morphology in untreated Krt14-CKO mice. (E to G) Representative images and quantitative analysis of Krt14 protein expression in the urothelium of BBN-treated WT and Krt14-CKO mice at different time points. Data are expressed as the mean ± SD. The symbol (*) indicates a statistically significant decrease in *Krt14* expression in Krt14-CKO mice compared with WT controls under untreated conditions (p < 0.05).

**Fig.S7.** **Panoramic histological view of bladder tissues after 15 weeks of BBN treatment.** (A) Representative panoramic low-magnification HE images of bladder sections from WT and Krt14-CKO mice after 15 weeks of BBN treatment. Black boxes indicate the regions shown at higher magnification in Fig. 1Q.

**Fig.S8.** **Panoramic and magnified histological views of bladder tissues after 25 weeks of BBN treatment.** (A to B) Panoramic low-magnification HE images of bladder sections from all WT and Krt14-CKO mice analyzed in Fig. 1S (n = 14 per group). The left panels show panoramic views, and the right panels show magnified views of the boxed regions.

**Fig.S9. KRT14-mediated oncogenic role of IGF2BP1.** (A to C) Quantitative PCR analysis of *IGF2BP1* mRNA expression levels across experimental groups. (D to F) Western blot analysis of IGF2BP1 protein expression levels across experimental groups. (G to H) Representative images and quantitative analysis of Igf2bp1 protein expression in the urothelium of WT mice treated with BBN at different time points. (I) Correlation analysis between Krt14 and Igf2bp1 protein expression in the BBN-induced mouse model. Data are presented as the mean ± SD. The symbol (*) indicates a statistically significant difference (p < 0.05) in IGF2BP1 expression, observed both *in vitro* (upon KRT14 overexpression or knockout) and *in vivo* (between consecutive time points during BBN induction).

**Fig.S10. Pseudotime analysis reveals the role of Krt14-Igf2bp1 axis in BBN-induced BCa progression.** (A) t-SNE plot illustrating the distribution of distinct cell types identified from scRNA-seq data of bladder tissues from WT and Krt14-CKO mice under vehicle and BBN treatment conditions, with each cell type color-coded. (B) UMAP plot colored by signature genes expression among urothelial cell subtypes in WT mice (n = 3). (C) CytoTRACE2-based assessment of tumor cell developmental potential. (D) Pseudotime analysis of urothelial cell subtypes in bladder tumors from BBN-treated WT mice at 25 weeks post-induction, with subtype-specific trajectories visualized using Monocle3. (E) Differentiation trajectories of urothelial cell subpopulations. (F) t-SNE plot showing the expression of representative signature genes in urothelial subtypes from BBN-treated WT and Krt14-CKO mice.

**Fig.S11.** **Igf2bp1 expression in bladder tissues of Krt14-CKO and WT mice following BBN treatment.** (A to B) Quantitative analysis showing Igf2bp1 protein expression in the urothelium of BBN-treated Krt14-CKO and WT mice at different time points. Data are presented as the mean ± SD.

**Fig.S12. Functional validation of IGF2BP1 in regulating the invasiveness of cells with differential KRT14 expression.** (A to B) Western blot analysis of IGF2BP1 expression in KRT14-overexpressing BMIBC cells under different treatment conditions. (C) Western blot analysis of IGF2BP1 expression in KRT14-knockout J82 cells under different treatment conditions. (D to I) Representative images and quantitative analysis of migration and invasion abilities in BMIBC cells with differential KRT14 expression following IGF2BP1 rescue. Data are presented as the mean ± SD. The symbols (*) and (#) indicate statistically significant differences (p < 0.05). The symbol (*) denotes a significant change in the invasion ability of KRT14-overexpressing or KRT14-knockout cells compared with their respective controls, whereas (#) indicates the corresponding change observed following IGF2BP1 knockout or rescue.

**Fig.S13.** **Expression of squamous differentiation-associated markers in WT and Krt14-CKO tumors after 25 weeks of BBN treatment.** (A to F) Feature plots and violin plots showing the expression levels of squamous differentiation-associated genes in WT and Krt14-CKO tumors from mice treated with BBN for 25 weeks. Feature plots show the distribution of marker gene expression in epithelial cell populations, and violin plots show the corresponding expression levels in WT and Krt14-CKO tumors. Statistical significance was determined as indicated.

**Fig.S14.** **Cellular composition and immune-related signatures in WT and Krt14-CKO tumors after 25 weeks of BBN treatment.** (A) Proportional analysis showing the distribution of major cell populations identified by scRNA-seq in WT and Krt14-CKO tumors from mice treated with BBN for 25 weeks. (B to C) Violin plots showing the IFNγ score and T cell-inflamed GEP in WT and Krt14-CKO tumors. Statistical significance was determined as indicated.

**Fig.S15. Interaction between IGF2BP1 and KRT14 proteins.** (A) Co-IP assay confirming the physical interaction between KRT14 and IGF2BP1. (B to C) Endogenous Co-IP showing the interaction between KRT14 and IGF2BP1 in T24T and J82 cells. IgG was used as a negative control. (D) Schematic representation of the IGF2BP1 truncation constructs showing the RRM (1-156 aa) and KH1-4 domains (157-577 aa). (E) Identification of the specific structural domains responsible for the IGF2BP1-KRT14 interaction by Co-IP assays. (F) DMFold-predicted model illustrating the interaction between the N-terminal region of KRT14 (pink) and the KH1 domain of IGF2BP1 (cyan). (G) LocNES prediction of the NES in KRT14. (H) DMFold modeling of the GKEG-to-GAAG mutation within the KH2 domain of IGF2BP1 (cyan), illustrating the altered interaction with the N-terminal region of KRT14 (pink). (I) DMFold modeling of the LDEL-to-LAAL mutation within the NES motif of the KRT14 N terminus and its impact on binding to the IGF2BP1 KH2 domain (cyan).

**Fig.S16. The RNA-binding motif in the IGF2BP1 KH2 domain is critically regulated by KRT14.** (A to B) Representative images showing the migration and invasion abilities of U5637 (KRT14) and T24T (KRT14) cells following IGF2BP1 modulation. (C) Western blot analysis of J82 (KOKRT14) cells overexpressing IGF2BP1-WT or IGF2BP1-K2AA. (D to E) Representative images and quantitative analysis of migration and invasion in J82 (KOKRT14) cells expressing IGF2BP1-WT or IGF2BP1-K2AA. Data are expressed as the mean ± SD. The symbol (*) indicates a significant decrease in invasive capacity following IGF2BP1 knockout compared with control cells (p < 0.05). The symbol (#) indicates a significant increase in invasive capacity after rescue with IGF2BP1-WT compared with IGF2BP1-knockout cells (p < 0.05). The symbol (♠) indicates a significant decrease in invasive capacity after rescue with IGF2BP1-K2AA compared with IGF2BP1-WT rescue (p < 0.05).

**Fig.S17. Transcriptional regulation of IGF2BP1 by KRT14.** (A to B) Dual-luciferase reporter assays evaluating the effects of KRT14-WT and KRT14-MUT on IGF2BP1 promoter activity. (C) Schematic representation of the full-length IGF2BP1 transcript. (D to E) Expression levels of *let-7* in BMIBC cells across different experimental groups. (F to G) Functional validation of the regulatory effects of wild-type versus mutant KRT14 on IGF2BP1-Long-2 3′UTR activity using dual-luciferase reporter assays. Data are expressed as the mean ± SD and the symbol (*) and (#) indicate a significant difference at p < 0.05. (*) denotes reduced promoter activity in KRT14-WT versus control, and (#) denotes increased promoter activity in KRT14-MUT versus KRT14-WT.

**Fig.S18. Mechanism of IGF2BP1-mediated stabilization of its own mRNA.** (A) Validation of IGF2BP1 protein expression in BMIBC cells treated with increasing concentrations of exogenous IGF2BP1. (B to C) Analysis of endogenous *IGF2BP1* mRNA expression in BMIBC cells treated with increasing concentrations of exogenous IGF2BP1. (D to E) Assessment of endogenous *IGF2BP1* mRNA stability under the same conditions. (F to G) Dual-luciferase reporter assays analyzing endogenous IGF2BP1 3′UTR activity in BMIBC cells exposed to increasing concentrations of exogenous IGF2BP1. (H) Schematic representation of IGF2BP1 RNA-binding site mutations. Specifically, residues K213, E214, K294, and E295 were substituted with aspartic acid (D), while K423, K424, K505, and G506 were substituted with glutamic acid (E). (I) Validation of the expression of IGF2BP1 RNA-binding site mutants in U5637 cells by Western blot analysis. (J) Assessment of the binding affinity of IGF2BP1 to its own mRNA at different RNA-binding sites. Data are expressed as the mean ± SD. The symbol (*) indicates a statistically significant difference (p < 0.05) in endogenous IGF2BP1 expression or IGF2BP1-Long-3'UTR luciferase reporter activity among different concentrations of exogenous IGF2BP1 treatment, as well as a significant difference in binding affinity between IGF2BP1 point mutants and IGF2BP1-WT.

**Fig.S19.** **Analysis of *VCAN* and *ZEB1* mRNA expression and stability in KRT14-overexpressing T24T cells.** (A to B) Actinomycin D chase assays assessing the stability of *VCAN* and *ZEB1* mRNAs in T24T cells overexpressing KRT14 or vector control. (C to D) qPCR analysis of *VCAN* and *ZEB1* mRNA expression levels in T24T cells overexpressing KRT14 or vector control. Data are presented as mean ± SD. The symbol (*) indicates a significant increase in the indicated parameter in KRT14-overexpressing cells compared with control cells (p < 0.05).

**Fig.S20. D226 and E227 residues of KRT14 are essential for IGF2BP1 binding and functional regulation.** (A to D) Representative images and quantitative analysis of migration and invasion abilities of U5637 and T24T BMIBC cells expressing KRT14-WT or KRT14-MUT. (E) Western blot analysis of J82 (KOKRT14) cells overexpressing KRT14-WT or KRT14-MUT. (F to G) Representative images and quantitative analysis of migration and invasion abilities of J82 (KOKRT14) cells expressing KRT14-WT or KRT14-MUT. The symbol (*) indicates that KRT14-WT cells show significantly increased invasive capacities compared with control cells (p < 0.05). The symbol (#) indicates that KRT14-MUT cells show a significant decrease in invasive capacities compared with KRT14-WT cells (p < 0.05).

**Tables**

**Table S1.** List of cluster-specific marker genes identified in the GSE267718 scRNA-seq dataset of human BCa.

**Table S2.** List of genes corresponding to each group in the Venn diagram shown in Fig. 1G, derived from the integrative analysis of human and mouse epithelial gene sets.

**Table S3.** The information of BCa patients including case number, gender, age and clinical classification.

**Video S1.** The dynamic movement patterns of IGF2BP1 mRNA in U5637 (Vector) and U5637 (HA-KRT14) cells

**References**

[1]	C. Huang, X. Liao, H. Jin, F. Xie, F. Zheng, J. Li, C. Zhou, G. Jiang, X. R. Wu, C. Huang, *Mol Ther Nucleic Acids* **2019**, *16*, 51, <https://doi.org/10.1016/j.omtn.2019.01.014>.

[2]	W. Guo, Z. Yang, Q. Xia, J. Liu, Y. Yu, J. Li, Z. Zuo, D. Zhang, X. Li, X. Shi, C. Huang, *Cell Mol Life Sci* **2011**, *68* (3), 475, <https://doi.org/10.1007/s00018-010-0459-7>.

[3]	S. V. Vasaikar, A. P. Deshmukh, P. den Hollander, S. Addanki, N. A. Kuburich, S. Kudaravalli, R. Joseph, J. T. Chang, R. Soundararajan, S. A. Mani, *Br J Cancer* **2021**, *124* (1), 259, <https://doi.org/10.1038/s41416-020-01178-9>.

[4]	J. C. Thompson, W. T. Hwang, C. Davis, C. Deshpande, S. Jeffries, Y. Rajpurohit, V. Krishna, D. Smirnov, R. Verona, M. V. Lorenzi, C. J. Langer, S. M. Albelda, *Lung Cancer* **2020**, *139*, 1, <https://doi.org/10.1016/j.lungcan.2019.10.012>.

[5]	A. G. Robertson, J. Kim, H. Al-Ahmadie, J. Bellmunt, G. Guo, A. D. Cherniack, T. Hinoue, P. W. Laird, K. A. Hoadley, R. Akbani, M. A. A. Castro, E. A. Gibb, R. S. Kanchi, D. A. Gordenin, S. A. Shukla, F. Sanchez-Vega, D. E. Hansel, B. A. Czerniak, V. E. Reuter, X. Su, B. de Sa Carvalho, V. S. Chagas, K. L. Mungall, S. Sadeghi, C. S. Pedamallu, Y. Lu, L. J. Klimczak, J. Zhang, C. Choo, A. I. Ojesina, S. Bullman, K. M. Leraas, T. M. Lichtenberg, C. J. Wu, N. Schultz, G. Getz, M. Meyerson, G. B. Mills, D. J. McConkey, J. N. Weinstein, D. J. Kwiatkowski, S. P. Lerner, *Cell* **2018**, *174* (4), 1033, <https://doi.org/10.1016/j.cell.2018.07.036>.

[6]	M. Ayers, J. Lunceford, M. Nebozhyn, E. Murphy, A. Loboda, D. R. Kaufman, A. Albright, J. D. Cheng, S. P. Kang, V. Shankaran, S. A. Piha-Paul, J. Yearley, T. Y. Seiwert, A. Ribas, T. K. McClanahan, *J Clin Invest* **2017**, *127* (8), 2930, <https://doi.org/10.1172/jci91190>.
